# Matrix‑stiffness‑triggered mechanotransduction fuels hepatocellular carcinoma proliferation through Hippo‑orchestrated ANKRD1‑PRMT5 complex‑driven arginine methylome remodeling

**DOI:** 10.1016/j.mmr.2026.100075

**Published:** 2026-10-24

**Authors:** Jia-Cheng Huang, Bao-Dong Chen, Yuan Xu, Si-Jie Zheng, Yi-Qing Hu, Cai-Xu Pan, Li-Zhu Zhu, Ying Chi, Meng-Lan Wang, Fei Wang, Yi-Xin Hua, Peng-Yu Gong, Jian-Xiang Chen, Shu-Sen Zheng, Yi-Ting Qiao

**Affiliations:** a Division of Hepatobiliary and Pancreatic Surgery, Department of Surgery, The First Affiliated Hospital, Zhejiang University School of Medicine, Hangzhou 310003, China; b Zhejiang Provincial Key Laboratory of Anti-Cancer Chinese Medicines and Natural Medicines, Department of Hepatology, the Affiliated Hospital of Hangzhou Normal University, School of Pharmacy, Hangzhou Normal University, Hangzhou 311121, China; c NHC Key Laboratory of Combined Multi-organ Transplantation, Key Laboratory of Organ Transplantation, Hangzhou 310003, China; d Institute of Organ Transplantation, Zhejiang University, Hangzhou 310003, China; e Otolaryngology and Head and Neck Center, Cancer Center, Department of Head and Neck Surgery, Zhejiang Provincial People’s Hospital, Affiliated People’s Hospital, Hangzhou Medical College, Hangzhou 310014, China; f The Affiliated LiHuiLi Hospital of Ningbo University, Ningbo 315046, Zhejiang, China; g Edinburgh University Joint Institute, Zhejiang University, Haining 314400, Zhejiang, China

**Keywords:** Fibrosis, Hepatocellular carcinoma (HCC), Ankyrin repeat domain protein 1 (ANKRD1), Protein arginine methyltransferase 5 (PRMT5), Hippo pathway

## Abstract

**Background:**

Stiffened extracellular matrix is a primary risk factor for hepatocellular carcinoma (HCC). We aim to investigate the mechanism by which matrix stiffness promotes malignant proliferation and develop an experimental therapeutic strategy for HCC.

**Methods:**

CCl_4_-induced and ATP-binding cassette subfamily B member 4 (*Abcb4*)^-/-^ spontaneous liver-fibrosis mouse models were used to validate the tumour-promoting effect of matrix stiffness in HCC. Polyacrylamide gel was used to simulate matrix with varying stiffness. RNA sequencing (RNA-seq), *in vitro* proliferation assays, and *in vivo* tumour models were used to elucidate the role of mechanically responsive protein ankyrin repeat domain protein 1 (ANKRD1) in HCC. Biochemical assays were performed to reveal the interaction between ANKRD1 and protein arginine methyltransferase 5 (PRMT5). Chromatin immunoprecipitation and luciferase reporter assays were performed to confirm the pattern of transcriptional regulation. The anti-tumour efficiency of Hippo pathway inhibitors and the PRMT5 inhibitor was evaluated *in vitro* and *in vivo*.

**Results:**

Analysis of human hepatic fibrosis index, fibrotic mice bearing HCC, and polyacrylamide gel-based soft matrix culture model demonstrated that a stiff microenvironment promoted HCC progression. RNA-seq of HCC cells cultured on varying stiffness conditions identified ANKRD1 as a target for mechanical signaling, which was further validated in human HCC tissue samples at both RNA and protein levels. Gene function analysis revealed that ANKRD1 promoted HCC progression, which partially mediated stiffness-associated malignant proliferation. Mechanistically, protein-protein interaction assays and quantitative post-translational modification assays proved the interaction between the C-terminus of ANKRD1 and PRMT5, which promoted the cytoplasmic localization of PRMT5 and arginine methylome remodeling. Furthermore, inhibitors of the Hippo pathway decreased ANKRD1 expression and sensitized HCC cells to PRMT5 inhibitor LLY-283 in both an *in vitro* cytotoxicity assay and a murine fibrotic HCC model.

**Conclusions:**

ANKRD1 is a mechanically responsive protein that promotes the proliferation of HCC cells by regulating PRMT5 activity. The combination of Yes1-associated transcriptional regulator/tafazzin (YAP/TAZ) inhibitors and a PRMT5 inhibitor is a potential therapeutic strategy for HCC patients with a stiffened tumour microenvironment.

## 1 Background

Liver cancer is the second most lethal malignancy, causing approximately 830,000 deaths worldwide each year [1]. Hepatocellular carcinoma (HCC) constitutes the predominant subtype of primary liver cancer. Cirrhosis and fibrosis have long been recognized as critical risk factors for HCC, and the biological mechanisms underlying this causality have been investigated from various perspectives [2]. Hippo pathway, a mechanotransduction signalling pathway, maintains the upstream phosphorylation cascade of key tumour suppressors [e.g., mammalian sterile 20-like kinase 1/2 (MST1/2) and large tumor suppressor kinase 1/2 (LATS1/2)], and its inactivation results in the nuclear translocation of the downstream transcriptional cofactors Yes1-associated transcriptional regulator (YAP) and tafazzin (TAZ), leading to the subsequent expression of pro-proliferative and pro-survival genes [3], [4], [5]. Recently, small-molecule compounds against YAP/TAZ have been under intensive preclinical and clinical evaluation for the treatment of solid tumours as monotherapy or combination therapies [6], [7]. The mechanism by which the stiffened tumour microenvironment (TME) contributes to malignant behaviour in HCC, especially with respect to post-translational modifications, might provide guidance for the design of specific combination therapies for HCC patients with underlying fibrosis and cirrhosis.

Ankyrin repeat domain protein 1 (ANKRD1), a member of the ankyrin repeat domain protein family, is primarily distributed in cardiac and skeletal muscles subjected to tensile biophysical mechanical forces under normal physiological conditions, where it regulates cytoskeletal protein function through interactions with skeleton-related proteins, such as Tintin and Talin [8], [9]. Studies have shown that ANKRD1 is upregulated in ovarian cancer and closely associated with tumour chemoresistance [10], [11]. Previous investigations have identified ANKRD1 as a potential downstream target of the Hippo signalling pathway in mammary epithelial cells [12], [13]. However, it remains unclear whether ANKRD1 interacts with other proteins or exerts pro-tumorigenic functions in HCC tissues characterized by extracellular matrix (ECM) stiffening.

Protein arginine methyltransferase 5 (PRMT5) is a key methyltransferase involved in post-translational modifications for essential subunits of protein complexes that regulate gene expression, including histones, c-Myc, and several RNA-binding proteins [14], [15], [16]. Recently, several studies have identified PRMT5 as a promising therapeutic target for various types of solid tumours, including HCC, the genetic and pharmacological inhibition of which has shown significant anti-proliferative effects in a range of preclinical models [17], [18], [19]. Given the dysregulation of PRMT5 in various tumours and the robust efficacy demonstrated in preclinical studies targeting PRMT5, researchers have developed a variety of PRMT5 inhibitors with distinct targeting sites, several of which are currently in clinical trials [17], [20], [21]. Although PRMT5 protein expression generally tends to increase in tumour tissues, further studies have revealed that its subcellular localization is variable [22], [23]. For instance, a retrospective study on lung cancer tissues revealed that compared with nuclear PRMT5 expression, high cytoplasmic PRMT5 expression was more significantly correlated with poor prognosis, indicating that PRMT5 might exhibit varying pro-tumorigenic effects depending on its subcellular localization [24]. Another study on clinical HCC samples revealed a significant correlation between cytoplasmic PRMT5 and the pro-tumorigenic nuclear localization phenotype of β-catenin [25]. However, the mechanism underlying the cytoplasmic accumulation and hyperactivation of PRMT5 has not yet been fully elucidated, and it is unclear whether stiffened TME contributes to the regulation of PRMT5 function in HCC.

This study aimed to explore the mechanism by which matrix stiffness promotes malignant proliferation in HCC and develop an experimental therapeutic strategy for a fibrotic HCC model. First, both *in vitro* and *in vivo* models were employed to demonstrate the pro-proliferative effect of matrix stiffness in HCC. *ANKRD1* was characterised as a pivotal gene governing mechanotransduction‑associated malignancy, which was further validated in multiple models. Then, both upstream and downstream oncogenic mechanisms involved in matrix‑stiffness‑triggered ANKRD1 overexpression were investigated by biochemical analysis, and its translational potential was evaluated by small-molecule compound-mediated targeted inhibition.

## 2 Methods

### 2.1 Determination of the hepatic fibrosis index

The hepatic fibrosis index was evaluated based on the Cancer Genome Atlas Liver Hepatocellular Carcinoma (TCGA-LIHC) cohort. Specifically, the hepatic fibrosis-related gene set “HP_HEPATIC_FIBROSIS” was downloaded from the Molecular Signatures Database (https://www.gsea-msigdb.org/gsea/msigdb/index.jsp) (Additional file 1: Table S1). The gene set variation analysis (GSVA) package was applied to calculate the hepatic fibrosis index based on the HP_HEPATIC_FIBROSIS gene matrix transposed (GMT) file and TCGA-LIHC expression matrix [26]. For survival analysis, patients in the TCGA-LIHC cohort were stratified into three groups (high, medium, and low) based on the quartile values of the hepatic fibrosis index: the top 25% for high, the bottom 25% for low, and the rest for medium. The log-rank test was used to compare the prognosis between the high and low groups based on patients dichotomized into the top and bottom quartiles, and Kaplan-Meier survival curves were plotted. Pearson correlation analysis was performed to determine the relationship between ANKRD1 expression and the hepatic fibrosis index.

### 2.2 HCC sample collection

Experiments involving HCC tissue samples, including 75 in-house frozen and paraffin-embedded HCC tissues, were reviewed by the Clinical Research Ethics Committee of the First Affiliated Hospital, Zhejiang University School of Medicine (2025B-1081). The HCC specimens encompassed patients with tumour stages Ⅰ to Ⅳ. Written informed consent was obtained from all the subjects. Resected HCC tissues were obtained from the First Affiliated Hospital, Zhejiang University School of Medicine between January and December 2025. One portion of each surgically resected sample was stored at –80 °C, and the other portion was fixed with formalin. All the research procedures strictly adhered to the Declarations of Helsinki and Istanbul.

### 2.3 Animal models

The animal experiments were reviewed by the Institutional Animal Care and Use Committee of the First Affiliated Hospital Zhejiang University School of Medicine (2025137). C57BL/6 mice (N000013), BALB/c nude mice (D000521) and ATP-binding cassette subfamily B member 4 (*Abcb4*)^*-/-*^ mice were purchased from GemPharmatech (Nanjing, China). The number of animals used in each experiment was provided in the figure legends. This experimental animal modelling method guaranteed the survival of all the mice prior to experimentation, and the tumour burden at the experimental endpoint complied with animal ethics requirements.

For the CCl_4_-induced murine fibrosis model, CCl_4_ was diluted fourfold in corn oil and administered via intraperitoneal injection to 8-week-old male C57BL/6 mice (2 ml/kg). CCl_4_ was injected once a week, whereas twice-weekly injections were administered to establish advanced fibrosis. The treatment period lasted for 4 weeks in both groups. *Abcb4*^*-/-*^ mice (T007421) were used as the spontaneous liver fibrosis mouse model [27].

For the orthotopic murine HCC model, murine fibrosis C57BL/6 mice and *Abcb4*^*-/-*^ mice were anaesthetized, and Hep-53.4 cell (Meisen Chinese Tissue Culture Collections) suspension (e.g., 1×10^6^ cells in 10 μl mixture containing equal volume of fetal bovine serum (FBS)-free medium and Matrigel) was injected into C57BL/6 mice beneath the liver capsule using a microsyringe. those from wild type (WT) C57BL/6 mice and *Abcb4*^*-/-*^ normal livers without fibrosis were considered controls. The mice were sacrificed approximately 3 weeks after orthotopic inoculation.

For the subcutaneous xenograft tumour model, 2×10^6^ parental or *ANKRD1*-knockout HCCLM3 cells (China Center for Culture Collection) were resuspended in 100 μl of FBS-free medium and subcutaneously inoculated into BALB/c nude mice, and mice receiving WT HCCLM3 cells were used as controls. The mice were sacrificed approximately 3 weeks after subcutaneous inoculation.

For the subcutaneous fibrosis-related HCC xenograft tumour model, 5×10^6^ HCCLM3 cells and 3×10^7^ LX-2 cells (China Center for Culture Collection) were premixed and subcutaneously injected into BALB/c nude mice. The size of the subcutaneous xenograft tumour was monitored using a Vernier calliper every two days. The mice were sacrificed approximately 3 weeks after modelling.

### 2.4 Polyacrylamide gel-based soft matrix culture

A polyacrylamide gel solution containing 40% acrylamide (Thermo Fisher Scientific, USA) and 2% Bis solution (Bio-Rad, USA) was prepared at the desired ratio and polymerized with 0.1% ammonium persulfate (Aladdin, China) and 0.1% N,N,N’,N’-tetramethylethylenediamine (TEMED) (Aladdin, China). The formulation consisting of 40% acrylamide and 2% Bis solution in hydrogel substrates with different mechanical properties was described in a previously published study [28]. The hydrogel was then cut into appropriately sized circles and placed in a culture dish. 0.5 mmol/L Sulfo-SANPAH (Sigma, USA) was pipetted into the wells and irradiated with UV light at a wavelength of 365 nm for 1 h. After being washed with PBS, the polyacrylamide gel was coated with type I collagen (Solarbio, China) according to the manufacturer’s instructions. After being washed with PBS, the cells were seeded on the substrates with varying stiffness (e.g., 2, 8, 16, and 34 kPa).

### 2.5 Cytoplasmic and nuclear protein extraction

A nuclear and cytoplasmic protein extraction kit (Beyotime, China) was used to fractionate the nucleocytoplasmic proteins. The cells were harvested and washed with PBS. To obtain cytoplasmic proteins, 200 μl of cytoplasmic protein extraction reagent A containing protease and phosphatase inhibitors was added, followed by vigorous vortexing for 5 s and incubation for 15 min on ice. Cytoplasmic protein extraction reagent B (10 μl) was added, and the mixture was vigorously vortexed for 5 s and incubated on ice for 1 min. To obtain cytoplasmic proteins and nuclear pellets, the mixture was centrifuged at 15,000× *g* for 5 min. The supernatant was carefully pipetted into a new clean tube, and the nuclear pellet was washed three times with PBS. Nuclear protein extraction reagent (50 μl) containing protease and phosphatase inhibitors was added, and the samples were vortexed for 30 s. The samples were then placed on ice for 30 min and subsequently sonicated. After centrifugation at 15,000× *g* for 15 min, the supernatant containing the nuclear protein was transferred to a new tube. A BCA protein assay was performed to determine the concentrations of cytoplasmic and nuclear proteins.

### 2.6 PRMT methyltransferase type II specific activity assay

Type II PRMT activity was measured using an Epigenase PRMT Methyltransferase (Type II-Specific) Activity/Inhibition Assay Kit (EpigenTek, USA). The cytoplasmic protein was extracted and added to a microplate well coated with a type II PRMT substrate and covered with adhesive film. Blank and standard wells were included. The samples were incubated at 37 °C for 2 h. The reaction buffer was removed, and the wells were washed three times. The capture antibody solution was then added for incubation at room temperature for 1 h. Each well was washed three times and incubated with the detection antibody at room temperature for half an hour. After the wells were washed three times, 100 μl of developer solution was added for incubation at room temperature for 5 min. Then, 100 μl of stop solution was added, and the absorbance at 450 and 655 nm was measured. PRMT activity was calculated according to the manufacturer’s instructions.

### 2.7 Construction of an *ANKRD1*-knockout cell line

*ANKRD1-*knockout cell line was constructed by Obio Technology Co., Ltd. (Shanghai, China). In brief, one pair of sgRNAs was cloned and inserted into a lentivirus-v2-cas9 vector, and the plasmid was chemically transfected into HCCLM3 cells, which were subsequently cultured for 48 h. Puromycin was added to eradicate untransfected cells. The limiting dilution method was used to isolate monoclonal cells, followed by expansion. PCR, gel electrophoresis, and Sanger sequencing were performed to confirm *ANKRD1* knockout.

### 2.8 Other materials and methods

The methods of other experiments, including cell culture, chemicals and plasmids, Sirius Red staining, Western blotting analysis, immunohistochemical (IHC) staining, cell cycle analysis, real-time quantitative polymerase chain reaction (RT-qPCR), Cell counting kit-8 (CCK-8) assay, colony formation assay, 5-ethynyl-2’-deoxyuridine (EdU) proliferation assay, immunoprecipitation (IP) and mass spectrometry (MS) analysis, immunofluorescence (IF) staining, dual-luciferase reporter assay, RNA sequencing (RNA-seq), and chromatin immunoprecipitation (ChIP), were detailedly described in Additional file 1: Methods and Table S2.

### 2.9 Statistical analysis

Continuous variables that passed the normality test were presented as the mean±standard deviation (SD). Student’s *t*-test was used to compare the means between the two groups. Welch’s *t*-test was applied to compare two variables that passed the normality test with different SDs. Unsupervised clustering analysis of fibrosis-related gene expression was performed to categorize HCC patients with different fibrosis backgrounds. To estimate the synergistic effect for the combination of ETS-003 and LLY-283, the Bliss excess was calculated by subtracting the predicted cell viability from the observed cell viability [29]. Statistical analysis was performed using R version 4.5.1 or GraphPad. Statistical significance was set at *P*<0.05.

## 3 Results

### 3.1 Fibrotic TME promotes the malignant progression of HCC

To determine whether fibrotic TME was associated with poor prognosis for patients with HCC, the TCGA-LIHC cohort was scored and categorized into high-, medium-, and low-fibrosis groups according to the gene expression index of the “HP_HEPATIC_FIBROSIS” gene set (Fig. 1**a;** Additional file 1: Table S1). Log-rank analysis demonstrated that HCC patients with a high fibrosis signature had significantly shorter overall survival and progression-free intervals (Fig. 1**b**). Moreover, Sirius Red staining revealed that high-stage HCC tissues (stage Ⅲ and Ⅳ) contained more collagen than early-stage HCC tissues (stage Ⅰ and Ⅱ) did (Additional file 1: Fig. S1a).

**Fig. 1:**
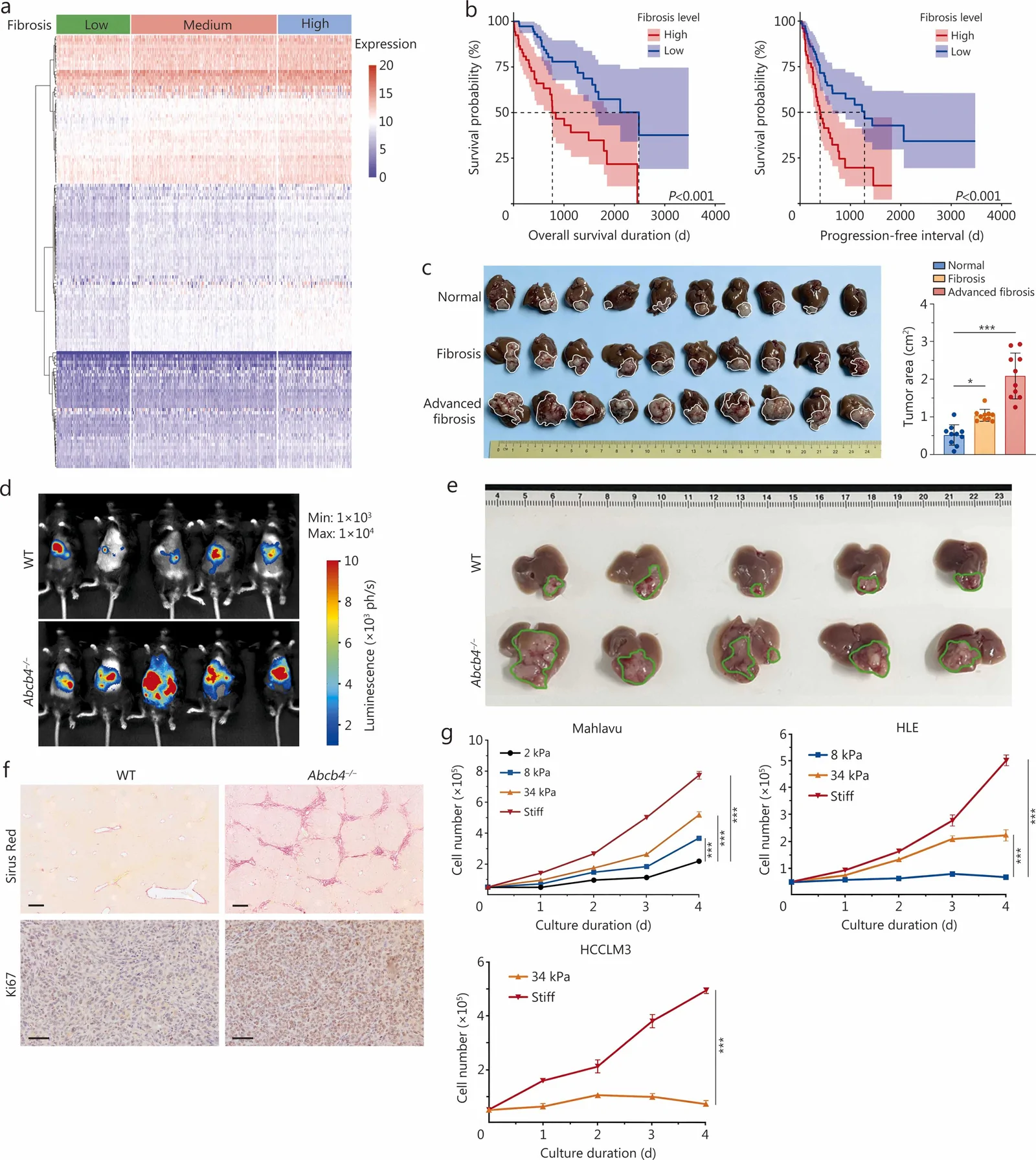
Fibrotic TME promotes malignant progression of HCC. **a** Heatmap showing the expression pattern of the “HP_HEPATIC_FIBROSIS” gene set in the TCGA-LIHC cohort. **b** Kaplan-Meier plots showing overall survival duration and progression-free interval for patients with high or low levels of fibrosis in the TCGA-LIHC cohort. **c** Image of resected livers from the orthotopic HCC model established in C57BL/6 or CCl_4_-induced liver fibrosis mice, and the quantified tumour area in each group (*n*=10). *t*-test was performed using “Normal” as control for each treatment. **d***In situ* fluorescence imaging results of the orthotopic HCC model established in WT (*n*=5) and *Abcb4*^-/-^ mice (*n*=5). **e** Image of resected livers from WT (*n*=5) and *Abcb4*^-/-^ mice (*n*=5) with orthotopic HCC tumours. **f** Sirius Red staining of liver tissues and Ki67 IHC staining of tumour tissues from WT and *Abcb4*^-/-^ mice. Scale bar=200 μm (Sirius Red) and 50 μm (IHC). **g** Results of the cell proliferation assays for Mahlavu, HLE, and HCCLM3 cells on cell culture substrates with various stiffnesses (*n*=3). *t*-test was performed using the softest substrate as the control for each cell line. The values represent the mean±SD. ^⁎^*P*<0.05, ^⁎⁎⁎^*P*<0.001. TME. Tumour microenvironment; HCC. Hepatocellular carcinoma; TCGA-LIHC. The Cancer Genome Atlas Liver Hepatocellular Carcinoma; WT. Wild type; Abcb4. ATP-binding cassette subfamily B member 4; IHC. Immunohistochemistry; SD. Standard deviation.

To further demonstrate that the fibrotic TME was a causal factor for HCC progression, we constructed a murine fibrosis model by intraperitoneal injection of CCl_4_ and then established an orthotopic HCC model with the murine HCC cell line Hep-53.4 injected into the mice (Additional file 1: Fig. S1b). At the experimental endpoint, the volume of the HCC tumours increased significantly as the degree of fibrosis increased (Fig. 1**c**). Furthermore, Sirius Red staining of adjacent liver tissues confirmed that mice receiving intraperitoneal CCl_4_ injection twice weekly exhibited the most severe hepatic fibrosis, and the IHC staining results also revealed that, compared with HCC tissues from normal livers, those from fibrotic and advanced fibrotic livers contained significantly more Ki67^+^ cells (Additional file 1: Fig. S1c).

Moreover, orthotopic HCC models were generated by intrahepatic inoculation of luciferase-transfected Hep-53.4 cells into age-matched WT mice and *Abcb4*^-/-^ mice with pre-existing spontaneous liver fibrosis, and tumour progression was assessed thereafter (Additional file 1: Fig. S1d**, e**). The volume of orthotopic HCC tumours was markedly larger in *Abcb4*^*-/-*^ mice than in WT mice, as shown by fluorescent *in situ* imaging and photographs of dissected livers (Fig. 1**d, e**). Sirius Red staining of liver tissues demonstrated that *Abcb4*^*-/-*^ mice were accompanied by fibrosis, and Ki67 IHC analysis of adjacent tumour tissues revealed that the proportion of proliferating cells in HCC tissues significantly increased with increasing degree of fibrosis (Fig. 1**f**).

Moreover, multiple HCC cell lines (Mahlavu, HLE, and HCCLM3) were cultured on regular plastic petri dishes (labelled “Stiff”) or polyacrylamide-based soft cell culture substrates with various stiffness, and CCK-8 assays revealed that their cell numbers were significantly greater on surfaces with higher stiffness (Fig. 1**g)**. Similarly, cell cycle analysis revealed that HCC cells exhibited obvious arrest in the G1 phase when cultured on 8 kPa soft substrates (HLE: 62.68±1.64 vs. 44.82±0.90, *P*<0.001; Mahlavu: 55.33±0.92 vs. 37.32±1.36, *P*<0.001) (Additional file 1: Fig. S1f**, g**).

Taken together, our results from multiple research models and clinical samples demonstrate that a stiffened microenvironment promotes the malignant progression of HCC.

### 3.2 Fibrotic TME induces ANKRD1 expression in HCC cells

Several studies have shown that stiffened ECM leads to global alterations in the transcriptome of mammalian cells [30], [31], [32], [33], [34]. In the aforementioned experiments, we observed that the proliferation of Mahlavu and HLE cells was significantly attenuated on 8 kPa substrates compared with that on conventional plastic dishes, suggesting potential alterations in stiffness-dependent transcriptional events. Here, we conducted RNA-seq to identify genes significantly upregulated by stiff culture substrates in two HCC cell lines (Mahlavu and HLE), which were simultaneously risk genes for poor prognosis in the TCGA-LIHC cohort, and 208 common genes were identified as potential oncogenes whose expression was induced by a stiffened microenvironment (Fig. 2**a**). Among these genes, *ANKRD1* was chosen for further analysis based on its fold change in expression and signal intensity in both HCC cell lines in the scatter plots (Fig. 2**b**). *In vitro* experiments with HCC cell lines, including qPCR and Western blotting assays, demonstrated that both the RNA and protein levels of *ANKRD1* increased as the stiffness of the culture substrates increased (Fig. 2**c, d**; Additional file 1: Fig. S2a**, b**). Moreover, Western blotting analysis from the WT and *Abcb4*^-/-^ mice with orthotopic HCC tissues in the spontaneous liver-fibrosis mouse model revealed that compared with those from WT mice, the protein level of ANKRD1 significantly increased in both adjacent normal and HCC tissues resected from *Abcb4*^*-/-*^ mice (Fig. 2**e;** Additional file 1: Fig. S2c). Moreover, 75 in-house frozen HCC specimens were categorized into high- and low-fibrosis groups according to the expression levels of 8 key genes (e.g., *TIMP1*, *TIMP2*, *COL1A1*, *COL3A1*, *COL6A1*, *ACTA2*, *CCN2*, and *LOX*) involved in ECM stiffening [35], [36], [37], [38], [39], [40] (Fig. 2**f**). As expected, the mRNA level of *ANKRD1* was markedly elevated in HCC samples with a high fibrosis signature (Fig. 2**g**). Moreover, Sirius Red staining and IHC staining of in-house paraffin-embedded HCC specimens indicated that HCC tissues with denser collagen deposition tended to have a higher positive rate for ANKRD1 expression (Fig. 2**h**). Similarly, Pearson correlation analysis revealed that the mRNA level of *ANKRD1* was positively associated with the fibrosis index of the “HP_HEPATIC_FIBROSIS” gene set in the TCGA-LIHC cohort (Fig. 2**i**), and the box plot of the mean expression level of *ANKRD1* revealed its steady increase with increasing fibrosis degree in the TCGA-LIHC cohort (Fig. 2**j**).

**Fig. 2:**
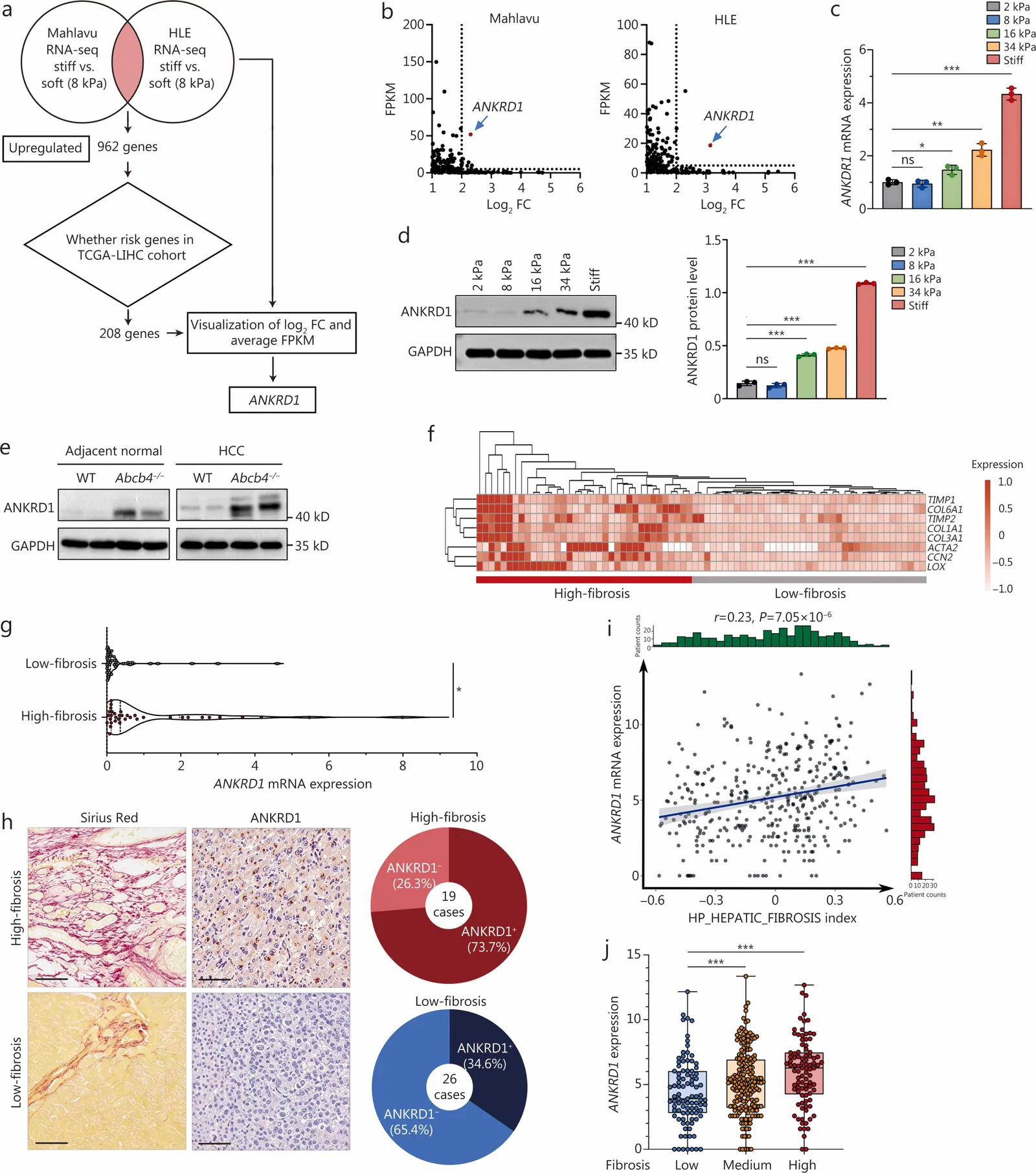
Fibrotic TME induces ANKRD1 expression in HCC. **a** Flowchart illustrating the process for screening key genes via whole-transcriptome sequencing. A plastic dish was used to mimic stiff substrates, and an 8 kPa polyacrylamide gel was used to mimic soft substrates. **b** Scatter plots showing the log_2_ FC and FPKM values of potential key genes in the RNA sequencing (RNA-seq) analysis of Mahlavu and HLE cells; *ANKRD1* is indicated with a red dot. qPCR (*n*=3) (**c**) and Western blotting (**d**) results showing ANKRD1 expression levels in Mahlavu cells cultured on substrates with various stiffnesses. *t*-test was performed using “2 kPa” as the calibrator for each group. The values represent the mean±SD. **e** Western blotting results showing ANKRD1 expression levels in HCC tissues and adjacent normal liver tissues from the WT and *Abcb4*^-/-^ mice with orthotopic HCC tumours. Heatmap (**f**) showing the expression levels of key genes involved in collagen deposition in 75 in-house frozen HCC samples; the *ANKRD1* expression level was determined by qPCR and visualized as a violin plot (**g**) with individual points. **h** Representative images showing the results of Sirius Red and IHC staining for ANKRD1 in in-house paraffin-fixed HCC specimens (Scale bar=100 μm), and the corresponding pie charts illustrating the positive rates of ANKRD1 in HCC specimens with or without fibrosis features in the IHC staining assay (Fisher’s exact test *P*=0.016). **i** Pearson correlation analysis of the HP_HEPATIC_FIBROSIS index and *ANKRD1* expression level in the TCGA-LIHC cohort. **j** Box-and-whisker plots showing *ANKRD1* expression levels in patients categorized into various fibrosis degrees according to the HP_HEPATIC_FIBROSIS index for the TCGA-LIHC cohort. *t*-test was performed using “Low” as the control for each group. The values represent the mean±SD. ^⁎^*P*<0.05, ^⁎⁎^*P*<0.01, ^⁎⁎⁎^*P*<0.001, ns non-significant. TME. Tumour microenvironment; ANKRD1. Ankyrin repeat domain protein 1; HCC. Hepatocellular carcinoma; TCGA-LIHC. The Cancer Genome Atlas Liver Hepatocellular Carcinoma; FPKM. Fragments per kilobase of transcript per million mapped reads; FC. Fold change; RNA-seq. RNA sequencing; WT. Wild type; Abcb4. ATP-binding cassette subfamily B member 4; GAPDH. Glyceraldehyde-3-phosphate dehydrogenase; COL6A1. Collagen type Ⅵ alpha 1 chain; TIMP1. TIMP metallopeptidase inhibitor 1; TIMP2. TIMP metallopeptidase inhibitor 2; COL1A1. Collagen type I alpha 1 chain; COL3A1. Collagen type III alpha 1 chain; ACTA2. Actin alpha 2, smooth muscle; CCN2. Cellular communication network factor 2; LOX. Lysyl oxidase; IHC. Immunohistochemistry; SD. Standard deviation.

Collectively, our results demonstrate that the fibrotic TME induces ANKRD1 expression in HCC via both *in vitro* and *in vivo* research models, as well as in clinical specimens.

### 3.3 ANKRD1 promotes the malignant progression of HCC

To determine whether ANKRD1 plays an oncogenic role in HCC, both *in vitro* and *in vivo* functional analyses were performed. First, ANKRD1 expression was measured by Western blotting in a range of HCC cell lines. The Western blotting results showed that ANKRD1 was detected in 7 out of 12 HCC cell lines (Additional file 1: Fig. S3a). The protein level of ANKRD1 was significantly suppressed when siRNA was transfected compared to the negative control (Additional file 1: Fig. S3b). CCK-8 assays were performed in Mahlavu and SNU-886 cells with siANKRD1, and both cell lines exhibited significant growth suppression after siANKRD1 treatment on stiff substrates (Fig. 3**a**). Similarly, siANKRD1 markedly reduced the colony numbers in the foci formation assay in both cell lines when they were cultured on stiff substrates (Fig. 3**b**), whereas the results of the EdU staining assay revealed that the percentage of proliferating cells was reduced by approximately 50% after *ANKRD1* silencing (Fig. 3**c**). Moreover, cells with *ANKRD1* knockdown exhibited markedly lower cell numbers compared with control cells on stiff substrates, while did not result in a remarkable change in cell numbers on 8 kPa after 48 h (Fig. 3**d**). Vice versa, ANKRD1 overexpression markedly increased the proliferation of Mahlavu cells when they were cultured on soft cell culture substrates (8 kPa), whereas these pro-proliferative effects were attenuated when the cells were cultured on stiff substrates, which might be attributed to increased endogenous expression of ANKRD1 in a stiff microenvironment (Additional file 1: Fig. S3c).

**Fig. 3:**
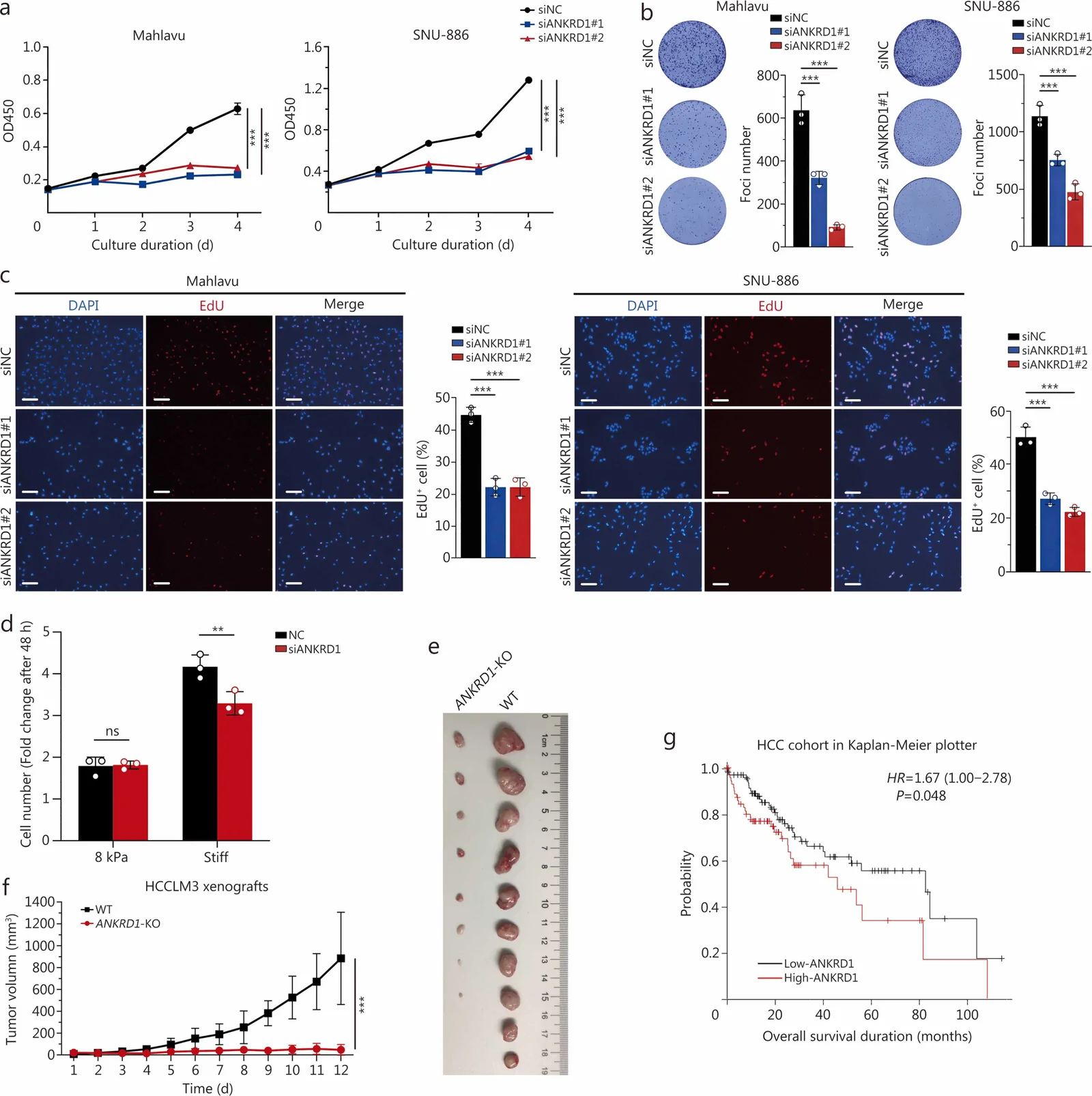
ANKRD1 promotes malignant progression of HCC. **a** Dot-line plots showing the results of the CCK-8 assay after *ANKRD1* silencing in Mahlavu and SNU-886 cells (*n*=3). **b** Representative images and bar charts quantifying the number of foci from the foci formation assays after *ANKRD1* was silenced in Mahlavu and SNU-886 cells cultured on stiff substrates (*n*=3). *t*-test was performed using “siNC” as the control for each group. **c** Representative fluorescence images (scale bar=50 μm) and bar charts quantifying the percentage of EdU^+^ cells from the EdU assay after *ANKRD1* was silenced in Mahlavu and SNU-886 cells (*n*=3). *t*-test was performed using “siNC” as the control for each group. **d** Grouped bar chart showing the fold change in cell number after *ANKRD1* was knocked down in Mahlavu cells (*n*=3). Images of resected tumour tissues (**e**) and dot-line plot showing tumour volume growth (**f**) from parental HCCLM3 cells and *ANKRD1*-KO cells (*n*=10). Tumor weight of each group was not measured as a visually obvious and significant difference in tumor volume was observed. **g** Kaplan-Meier survival analysis of patients with high or low ANKRD1 expression in the HCC cohort in the Kaplan-Meier plotter database (*P*=0.048). The values represent the mean±SD. ^⁎⁎^*P*<0.01, ^⁎⁎⁎^*P*<0.001, ns non-significant. ANKRD1. Ankyrin repeat domain protein 1; HCC. Hepatocellular carcinoma; OD. Optical density; CCK-8. Cell counting kit-8; NC. Negative control; DAPI. 4’,6-diamidino-2-phenylindole; EdU. 5-ethynyl-2’-deoxyuridine; WT. Wild type; KO. Knockout; SD. Standard deviation; *HR*. Hazard ratio.

In addition, a subcutaneous xenograft model was established to compare the oncogenicity of parental cells and HCCLM3 cells with Clustered Regularly Interspaced Short Palindromic Repeats (CRISPR) and CRISPR-associated protein 9 (Cas9)-mediated *ANKRD1* deletion (*ANKRD1-*KO) (Additional file 1: Fig. S3d**-f**). Both the images of the resected tumours and tumour volume curves demonstrated that *ANKRD1* deficiency significantly impaired the growth of HCC xenografts *in vivo* (Fig. 3**e, f**). Finally, Kaplan-Meier survival analysis revealed that high ANKRD1 expression was significantly correlated with a shortened overall survival duration for the HCC cohort in the Kaplan-Meier plotter database (Fig. 3**g**).

### 3.4 ANKRD1 interacts with the PRMT5 protein

To explore how ANKRD1 promoted HCC cell proliferation, MS-based proteomics was used to identify candidate proteins interacting with Flag-tagged ANKRD1, and interactome analysis revealed PRMT5 as a putative previously unreported ANKRD1-interacting partner (Fig. 4**a**). Given the established oncogenic role of PRMT5 in various malignancies [41], [42], [43], we next characterised the interaction between ANKRD1 and PRMT5 and its downstream biological effects.

**Fig. 4:**
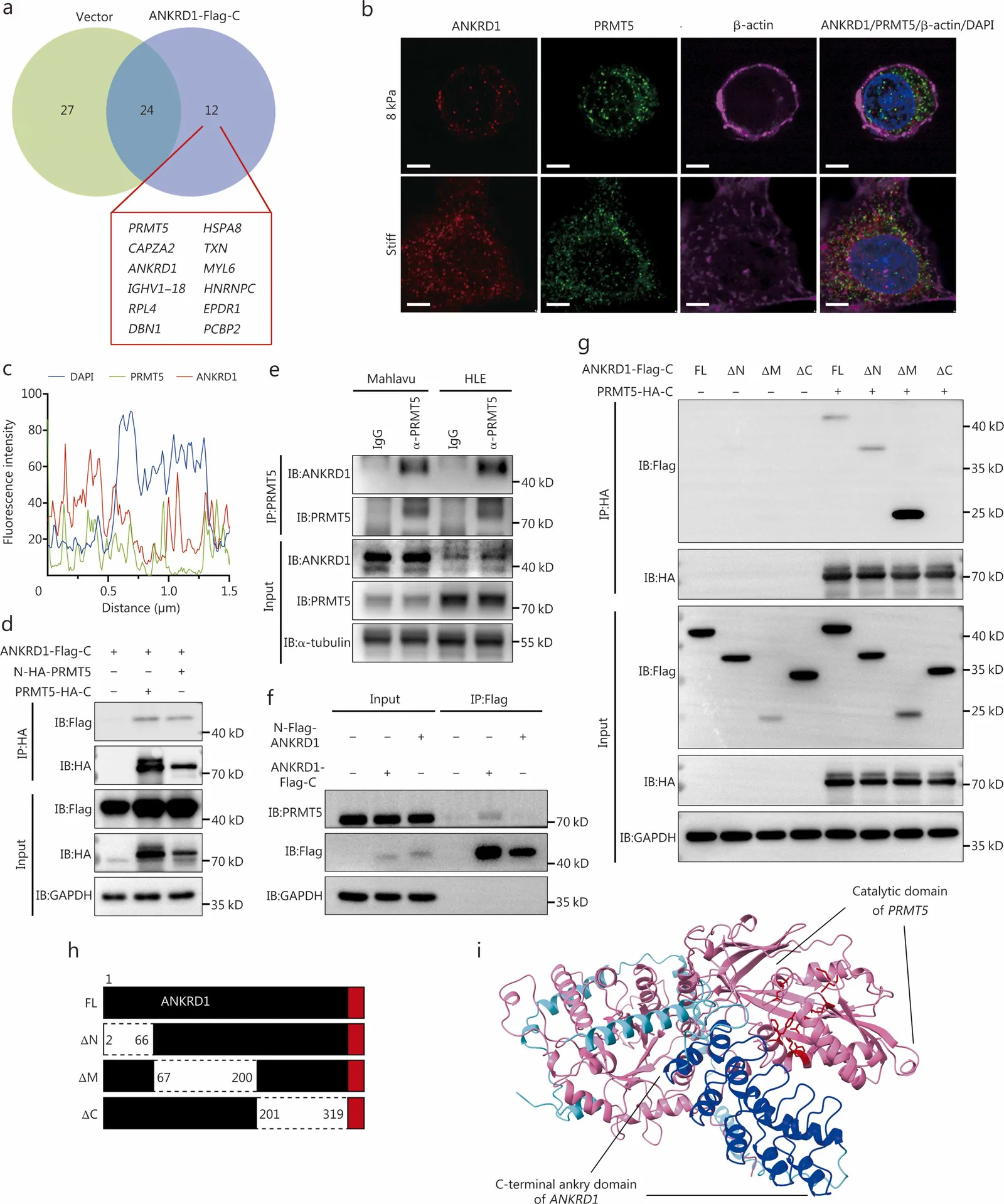
ANKRD1 interacts with PRMT5 protein. **a** Venn diagram summarizing the results of protein mass spectrometry analysis to identify potential proteins that interact with ANKRD1 in Mahlavu cells. **b** Representative fluorescence images showing the subcellular localization of ANKRD1 and PRMT5 in Mahlavu cells cultured on soft or stiff substrates. **c** Fluorescence intensity profile showing the co-localization of ANKRD1 and PRMT5 on stiff substrates. **d** Co-IP results for exogenously expressed ANKRD1 and PRMT5 in HEK-293T cells. **e** Endogenous co-IP results of ANKRD1 and PRMT5 in Mahlavu and HLE cells. **f** Semi-endogenous IP of exogenously expressed ANKRD1 and endogenous PRMT5 in Mahlavu cells. **g** Co-IP results for the exogenously expressed ANKRD1 truncation mutants and PRMT5 in HEK-293T cells. **h** Schematic showing the structures of the ANKRD1 truncation mutants. **i** Ribbon diagram of the protein interaction mode generated by AlphaFold3 for ANKRD1 (blue) and PRMT5 (pink), in which the anky domains of ANKRD1 were highlighted in navy, and the catalytic domain of PRMT5 was highlighted in red. ANKRD1. Ankyrin repeat domain 1; PRMT5. Protein arginine methyltransferase 5; HSPA8. Heat shock protein family A (Hsp70) member 8; CAPZA2. Capping actin protein of muscle Z-line subunit alpha 2; TXN. Thioredoxin; MYL6. Myosin light chain 6; IGHV1–18. Immunoglobulin heavy variable 1–18; HNRNPC. Heterogeneous nuclear ribonucleoprotein C; RPL4. Ribosomal protein L4; EPDR1. Ependymin related 1; DBN1. Drebrin 1; PCBP2. Poly(rC) binding protein 2; DAPI. 4’,6-diamidino-2-phenylindole; GAPDH. Glyceraldehyde-3-phosphate dehydrogenase; IP. Immunoprecipitation; IB. Immunoblotting; HA. Hemagglutinin; FL. Full; ΔC. C-terminal truncation; ΔM. Middle region truncation; ΔN. N-terminal truncation; Co-IP. Co-immunoprecipitation.

First, the distribution patterns of ANKRD1 and PRMT5 were analysed by IF imaging of Mahlavu cells cultured on both soft and stiff substrates. IF images revealed that ANKRD1 and PRMT5 were colocalized in the cytoplasm on stiff substrates (Fig. 4**b, c**). The interaction between ANKRD1 and PRMT5 was validated using a series of co-IP assays. In an exogenous co-IP experiment using HA-tagged PRMT5 as bait in HEK-293T cells, ANKRD1 could be immunoprecipitated by PRMT5 proteins tagged with HA at either the N- or C-terminus (Fig. 4**d**). Furthermore, the protein-protein interaction between ANKRD1 and PRMT5 was validated by endogenous co-IP experiments in both Mahlavu and HLE cells using an antibody against PRMT5 (α-PRMT5) (Fig. 4**e**). Interestingly, in a semi-endogenous IP assay in which Flag-tagged ANKRD1 was used as bait in Mahlavu cells, the endogenous PRMT5 protein could be precipitated only by the C-terminal Flag-tagged ANKRD1 but not by its N-terminal tagged counterpart (Fig. 4**f**), indicating that the N- and C-terminus of ANKRD1 might differ in terms of accessibility during the ANKRD1-PRMT5 interaction.

To determine the specific domain of ANKRD1 required for binding to PRMT5, N-terminal-, C-terminal-, and middle region-truncated constructs of ANKRD1 were generated and subjected to exogenous co-IP analysis in HEK-293T cells, and the ANKRD1-PRMT5 interaction was abolished when the C-terminal region (aa 201–319) of ANKRD1 was deleted (Fig. 4**g, h**), indicating that this region might be directly involved in the ANKRD1-PRMT5 interaction. In parallel, AlphaFold3’s multimer prediction mode was also employed to model the interaction between ANKRD1 and PRMT5, and the structural modelling suggested the spatial association between the C-terminal ankyrin repeat domains of ANKRD1 and the catalytic domain of PRMT5 (Fig. 4**i;** Additional file 2: Video), which was consistent with the phenomenon observed in the aforementioned co-IP assay of truncated ANKRD1 constructs. Collectively, our results demonstrate the protein-protein interaction between PRMT5 and the C-terminus of ANKRD1.

Supplementary material related to this article can be found online at doi:10.1016/j.mmr.2026.100075.

The following is the Supplementary material related to this article Video S1.

Video S1 Interaction between ANKRD1 and PRMT5 predicted by AlphaFold

### 3.5 ANKRD1 promotes the methyltransferase activity of PRMT5 in the cytoplasm

Given that ANKRD1 was predominantly distributed in the cytoplasm, we hypothesized that the ANKRD1-PRMT5 interaction might enhance the distribution of PRMT5 in the cytoplasm. To determine the influence of ANKRD1 expression on the subcellular localization of PRMT5, nuclear-cytoplasmic fractionation experiments were performed on Mahlavu and SNU-886 cells after *ANKRD1* overexpression or knockdown. Full-ANKRD1 overexpression led to a marked increase in the protein level of PRMT5 in the cytoplasm, but this effect was weaker when the C-terminal-truncated form of ANKRD1 was expressed (Fig. 5**a;** Additional file 1: Fig. S4a**, b**). PRMT5 methyltransferase activity of the cytoplasmic extracts indicated that overexpression of full-ANKRD1 increased cytoplasmic PRMT5 methyltransferase activity, but this effect was significantly reduced upon overexpression of the C-terminal-truncated clone (Fig. 5**b**). Moreover, overexpression of full-ANKRD1 led to a significant increase in the cell numbers of Mahlavu cells cultured on soft substrates (8 kPa), which was weakened with the C-terminal-truncated clone (Fig. 5**c**). On the other hand, in Mahlavu cells cultured on stiff substrates, overexpression of full‑ANKRD1 significantly accelerated cell proliferation, whereas overexpression of the C‑terminally truncated ANKRD1 exerted no obvious effect on HCC cell growth (Fig. 5**c**). Conversely, siANKRD1 resulted in significantly decreased PRMT5 levels in the cytoplasm of Mahlavu and SNU-886 cells (Fig. 5**d;** Additional file 1: Fig. S4c). Representative IF images of Mahlavu cells also indicated reduced cytoplasmic localization of PRMT5 after siANKRD1 was transfected (Fig. 5**e;** Additional file 1: Fig. S4d). Moreover, PRMT5 methyltransferase activity in the cytoplasm significantly decreased after siANKRD1 transfection (Fig. 5**f**). Therefore, these data demonstrate that ANKRD1 expression promotes the cytoplasmic localization and methyltransferase activity of PRMT5 in HCC cells.

**Fig. 5:**
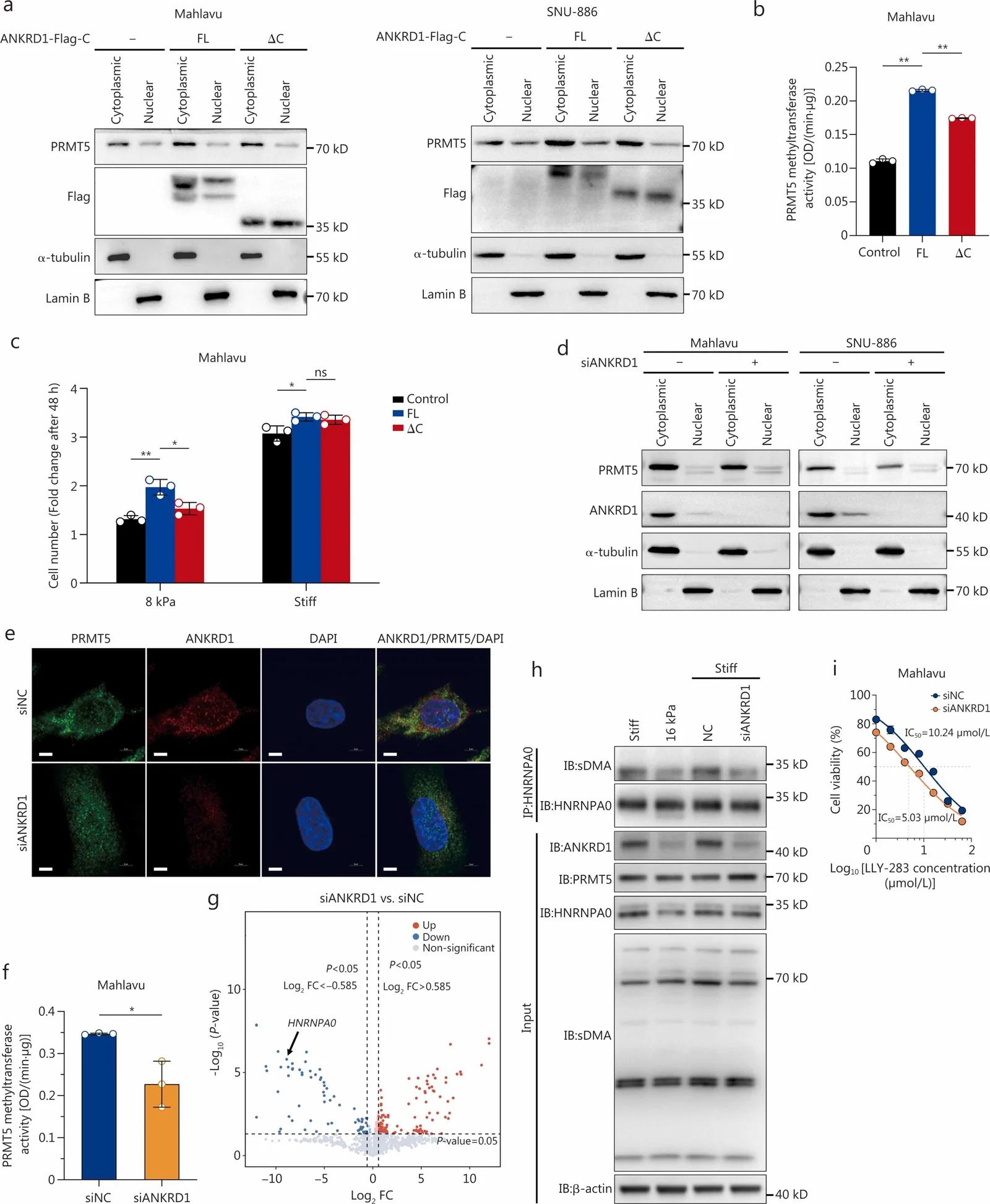
ANKRD1 promotes methyltransferase activity of PRMT5 in the cytoplasm. **a** Western blotting results of nuclear and cytoplasmic fractions from Mahlavu and SNU-886 cells overexpressing full-length ANKRD1 or the ∆C truncation mutant cultured on soft substrates. **b** Bar chart showing the results of the PRMT5 methyltransferase activity assay for the cytoplasmic lysates of Mahlavu cells overexpressing full-length ANKRD1 or the ∆C truncation mutant cultured on soft substrates (*n*=3). *t*-test was performed using “full” as control for each group. **c** Grouped bar chart showing the fold change in the number of Mahlavu cells overexpressing full-length ANKRD1 or the ∆C truncation mutant cultured on either soft or stiff substrates (*n*=3). *t*-test was performed using “full” as the control for 8 kPa and stiff culture conditions. **d** Western blotting results of nuclear and cytoplasmic fractions from Mahlavu and SNU-886 cells cultured on stiff substrates after *ANKRD1* was silenced. **e** Representative fluorescence images showing the subcellular localization of PRMT5 in Mahlavu cells cultured on stiff substrates after *ANKRD1* was knocked down. Scale bar=5 μm. **f** Bar chart showing the results of the PRMT5 methyltransferase activity assay of the cytoplasmic lysates of Mahlavu cells cultured on stiff substrates after *ANKRD1* was knocked down (*n*=3). **g** Volcano plot showing significantly altered proteins in the immunoprecipitates targeting sDMA modification via Pro-DIA LC-MC/MS. Red dots in the plot represent proteins with upregulated sDMA modification, while blue dots indicate proteins with downregulated sDMA modification. HNRNPA0 was indicated by the arrow in the plot. **h** Western blotting results of immunoprecipitates targeting HNRNPA0. **i** Drug toxicity dose-response curves of LLY-283 in the presence or absence of siRNA against ANKRD1 in Mahlavu cells cultured on stiff substrates. The values represent the mean±SD. ^⁎^*P*<0.05, ^⁎⁎^*P*<0.01, ns non-significant. ANKRD1. Ankyrin repeat domain 1; PRMT5. Protein arginine methyltransferase 5; DAPI. 4’,6-diamidino-2-phenylindole; FC. Fold change; IP. Immunoprecipitation; IB. Immunoblotting; NC. Negative control; sDMA. Symmetric dimethylarginine; HNRNPA0. Heterogeneous nuclear ribonucleoprotein A0; FL. Full; ΔC. C-terminal truncation; Co-IP. Co-immunoprecipitation; IC_50_. Half-maximal inhibitory concentration; SD. Standard deviation.

According to published studies on the pro-oncogenic role of PRMT5, many proteins require a symmetric dimethylarginine (sDMA) modification catalysed by PRMT5 to transform into their active forms with specific biological functions [19], [44], [45], [46]. Therefore, we speculated that stiffness-induced ANKRD1 expression might influence the sDMA modification pattern in HCC cells, especially for proteins that require sDMA modification in the cytoplasm. Quantitative proteomic analysis of anti-sDMA antibody immunoprecipitates revealed that siANKRD1 markedly decreased the sDMA modification of heterogeneous nuclear ribonucleoprotein A0 (HNRNPA0) (Fig. 5**g;** Additional file 1: Fig. S4e), which was consistent with previous PRMT‑methylome profiling that identified HNRNPA0 as a PRMT5 substrate [47]. Further IP validation demonstrated that the sDMA level of HNRNPA0 in Mahlavu cells was significantly reduced by either soft culture substrates (16 kPa) or *ANKRD1* knockdown (Fig. 5**h**). Additionally, individual knockdown of either *ANKRD1* or *HNRNPA0* markedly impaired the proliferation of Mahlavu HCC cells. However, simultaneous knockdown of these two genes produced no additional anti-proliferative effect (Additional file 1: Fig. S4f), indicating that these two genes might regulate cell proliferation through a shared signalling pathway. Moreover, *ANKRD1* knockdown reduced the half-maximal inhibitory concentration (IC_50_) of LLY-283, a PRMT5 inhibitor, in SNU-886 and Mahlavu cells (Fig. 5**i;** Additional file 1: Fig. S4g). Given the potential regulatory roles of ANKRD1 and HNRNPA0, as well as the critical functions of HNRNP family proteins in RNA splicing [48], we performed an analysis of splicing events in *ANKRD1*-knockout cell lines. After *ANKRD1* was knocked out, multiple types of differential splicing events were observed, including 1023 upregulated and 791 downregulated exon-skipping (SE) events (Additional file 1: Fig. S4h).

To further reveal the regulatory relationship between the stiffness/ANKRD1 axis and PRMT5 hyperactivation, Mahlavu cells overexpressing PRMT5 were cultured on either 16 kPa or stiff substrates for 48 h. PRMT5 overexpression resulted in a significant increase in cell number on 16 kPa substrates but not on stiff substrates, suggesting the pro-proliferative effect of endogenous PRMT5 might have reached saturation on stiff substrates (Additional file 1: Fig. S4i). Similarly, overexpression of PRMT5 in *ANKRD1*-knockdown cells remarkably mitigated the inhibitory effects of HCC proliferation, indicating that PRMT5 overexpression could partially reverse the cell proliferation suppression induced by siANKRD1 (Additional file 1: Fig. S4j).

Taken together, our data demonstrate that ANKRD1 promotes the cytoplasmic localization and methyltransferase activity of PRMT5 via protein-protein interactions, thus influencing the PRMT5 methylome in HCC cells, as indicated by the level of sDMA modification of HNRNPA0.

### 3.6 Stiffness-induced ANKRD1 expression is mediated by the Hippo pathway in HCC cells

Hippo pathway plays an important role in mechanical force-induced transcriptional adaptation, and previous investigations have identified ANKRD1 as a potential downstream target of the Hippo signalling pathway [12], [13]. To determine whether stiffness-induced ANKRD1 expression was mediated by the Hippo pathway, we applied siRNAs targeting YAP and TAZ, two critical effectors of the Hippo pathway, in Mahlavu and SUN-886 cells. Transfection of siRNA against *YAP* or *TAZ* significantly suppressed the expression of *YAP* or *TAZ*, respectively **(**Additional file 1: Fig. S5a**)**. qPCR analysis revealed that the mRNA level of *ANKRD1* was markedly reduced after *TAZ* silencing in both Mahlavu and SNU-886 cells (Fig. 6**a**). Notably, knockdown of *YAP* alone did not markedly inhibit ANKRD1 expression in either cell line, suggesting a preference for TAZ during ANKRD1 transcriptional regulation. Similarly, Western blotting analysis revealed that silencing either *YAP* or *TAZ* reduced the protein level of ANKRD1, whereas silencing *TAZ* had a more potent effect (Fig. 6**b;** Additional file 1: Fig. S5b). Moreover, three small-molecule inhibitors against YAP/TAZ-mediated transcription (GNE-7883, ETS-003, and ETS-006) were applied to a range of HCC cell lines, and both qPCR and Western blotting assays demonstrated that they could suppress *ANKRD1* expression at mRNA and protein levels in a dose-dependent manner (Fig. 6**c, d)**. These results indicate that *ANKRD1* is a target gene of the Hippo pathway in HCC cells.

**Fig. 6:**
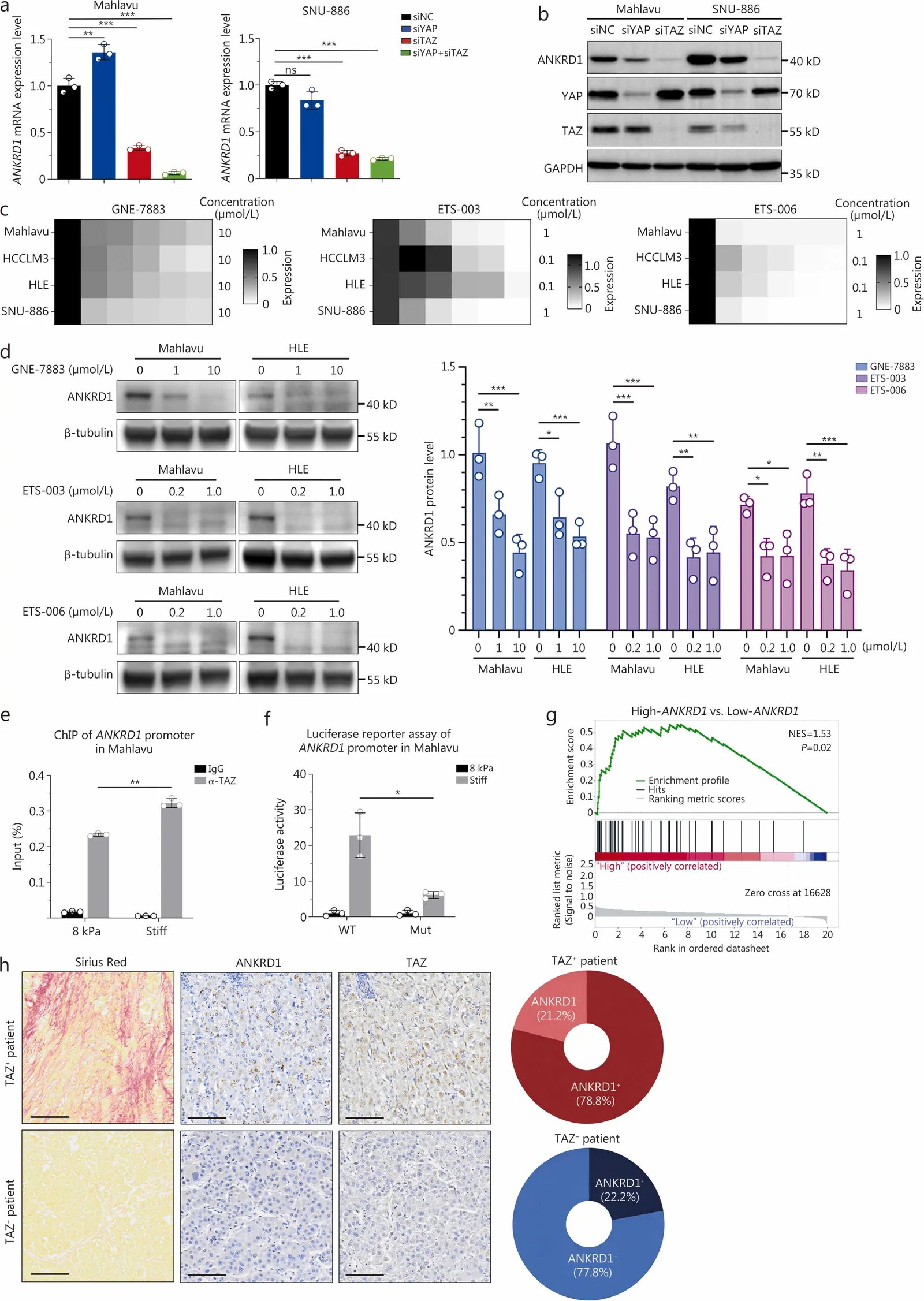
Stiffness-induced ANKRD1 expression is mediated by Hippo pathway in HCC. **a** Bar charts showing qPCR results measuring *ANKRD1* expression in Mahlavu and SNU-886 cells after knockdown of *YAP* or *TAZ* (*n*=3). *t*-test was performed using siNC as the control for each group. **b** Western blotting results for Mahlavu and SNU-886 cells after knockdown of *YAP* or *TAZ*. **c** Heatmaps visualizing ANKRD1 expression levels after treatment with GNE-7883, ETS-003, and ETS-006 in Mahlavu, HCCLM3, HLE, and SNU-886 cells. The highest concentration was listed in the figures, and the dilution factor was 2. **d** Western blotting results evaluating ANKRD1 expression levels after treatment with GNE-7883, ETS-003, and ETS-006 in Mahlavu and HLE cells. **e** Grouped bar chart showing the ChIP assay measuring the enrichment level of the *ANKRD1* promoter by the antibody against TAZ in Mahlavu cells cultured on soft or stiff substrates (*n*=3). **f** Grouped bar chart showing the results of the luciferase reporter assay of the wild type (WT) or mutant (Mut) *ANKRD1* promoter in Mahlavu cells cultured on soft or stiff substrates (*n*=3). **g** GSEA of the “GOBP_HIPPO_SIGNALING” gene set for the TCGA-LIHC cohort categorized according to the ANKRD1 expression level. **h** Representative images showing the results of Sirius Red staining for in-house paraffin-embedded HCC specimens and IHC analysis of ANKRD1 and TAZ expression, and the corresponding pie charts visualizing the positive rates of ANKRD1 in HCC specimens with positive or negative TAZ staining in the IHC assay (Fisher’s exact test *P*<0.001). Scale bar= 100 μm. The values represent the mean±SD. ^⁎^*P*<0.05, ^⁎⁎^*P*<0.01, ^⁎⁎⁎^*P*<0.001, ns non-significant. ANKRD1. Ankyrin repeat domain 1; NC. Negative control; HCC. Hepatocellular carcinoma; YAP. Yes1 associated transcriptional regulator; TAZ. Tafazzin; GAPDH. Glyceraldehyde-3-phosphate dehydrogenase; ChIP. Chromatin immunoprecipitation; WT. Wild type; Mut. Mutant; GSEA. Gene set enrichment analysis; TCGA-LIHC. The Cancer Genome Atlas Liver Hepatocellular Carcinoma; IHC. Immunohistochemistry; SD. Standard deviation.

To clarify whether stiffness regulates the transcription of *ANKRD1* through the Hippo pathway, ChIP-qPCR and promoter luciferase reporter assays were used to compare the status of the *ANKRD1* promoter when Mahlavu cells were cultured on soft or stiff substrates. ChIP-qPCR revealed significantly increased TAZ binding to promoter region 1 (–500 to –1 bp) of ANKRD1 in Mahlavu cells cultured on stiff substrates compared with those cultured on soft substrates (8 kPa), whereas no significant difference was observed in other regions (Fig. 6**e**; Additional file 1: Fig. S5c**, d**). Additionally, the activity of the luciferase reporter driven by the WT *ANKRD1* promoter increased on stiff substrates compared to soft substrates, whereas this response to stiffness was severely weakened for the mutant *ANKRD1* promoter in which the TEAD consensus sequence was deleted (Fig. 6**f;** Additional file 1: Fig. S5e).

We then analysed whether there was any correlation between ANKRD1 expression and Hippo pathway activity in HCC tissues. Patients from the TCGA-LIHC cohort were categorized according to the *ANKRD1* mRNA level, and gene set enrichment analysis (GSEA) revealed that compared with HCC tissues with low *ANKRD1* expression, high *ANKRD1* mRNA expression was associated with an obvious gene expression pattern in the “GOBP_HIPPO_SIGNALING” gene set (Fig. 6**g**). Moreover, Pearson correlation analysis of the TCGA-LIHC cohort revealed a significant positive correlation between the RNA expression levels of *ANKRD1* and connective tissue growth factor (*CTGF*), a well-recognized target gene reflecting Hippo pathway activity in a wide range of tumours, including HCC (Additional file 1: Fig. S5f). IHC and Sirius Red staining assays of in-house paraffin-fixed HCC specimens revealed that tissues with densely deposited collagen (as shown by extensive red-stained area in Sirius Red staining) tended to have higher rates of ANKRD1 and TAZ signal positivity, and the probability of being positive for ANKRD1 was significantly greater in the TAZ^+^ group than in the TAZ^–^ group (Fisher’s exact test *P*<0.0001; Fig. 6**h**).

In addition, we investigated the pro-proliferative role of ANKRD1 in the Hippo pathway. Firstly, *ANKRD1* knockdown resulted in proliferation inhibition similar to that induced by Hippo pathway inhibitors ETS-003 and ETS-006 in Mahlavu cells (Additional file 1: Fig. S5g). Furthermore, we assessed the sensitivity of ANKRD1-overexpressing HCC cells to the Hippo pathway inhibitors ETS-003 and ETS-006. In Mahlavu cells, ANKRD1-overexpression led to higher viability than control cells under ETS-003 or ETS-006 treatment, indicating reduced drug sensitivity. Consistently, ANKRD1-overexpressing HLE exhibited increased IC_50_ values for both ETS-003 and ETS-006 (Additional file 1: Fig. S5h**, i**). These results suggested that reintroduction of ANKRD1 partially rescued the proliferation suppression caused by Hippo pathway inhibitors.

Collectively, these data demonstrate that stiffness-induced ANKRD1 expression is mediated by the Hippo pathway in HCC.

### 3.7 YAP/TAZ inhibitors increase the therapeutic efficacy of PRMT5 inhibitor in a fibrosis-related HCC xenograft model

Given that PRMT5 served as a valuable therapeutic target for cancer translational research and our data proved that matrix-stiffness-triggered ANKRD1 promoted PRMT5 cytoplasmic localization in HCC cells, we hypothesized that the TME stiffness might correlate with cytoplasmic PRMT5 accumulation in human HCC tissues. Therefore, IF staining was performed to analyse the correlation between collagen deposition and cytoplasmic PRMT5 levels in both HCC and matched normal tissues. As expected, compared with adjacent liver tissues, HCC tissues tended to exhibit increased collagen deposition and PRMT5 expression (Fig. 7**a**). Further analysis revealed that PRMT5 showed a tendency for cytoplasmic localization in high fibrotic areas, whereas it tended to show whole-cell distribution in regions with low fibrosis levels **(**Fig. 7**b**). Collectively, these data indicated that the fibrotic microenvironment frequently observed with HCC tumours is associated with an increased level of cytoplasmic PRMT5.

**Fig. 7:**
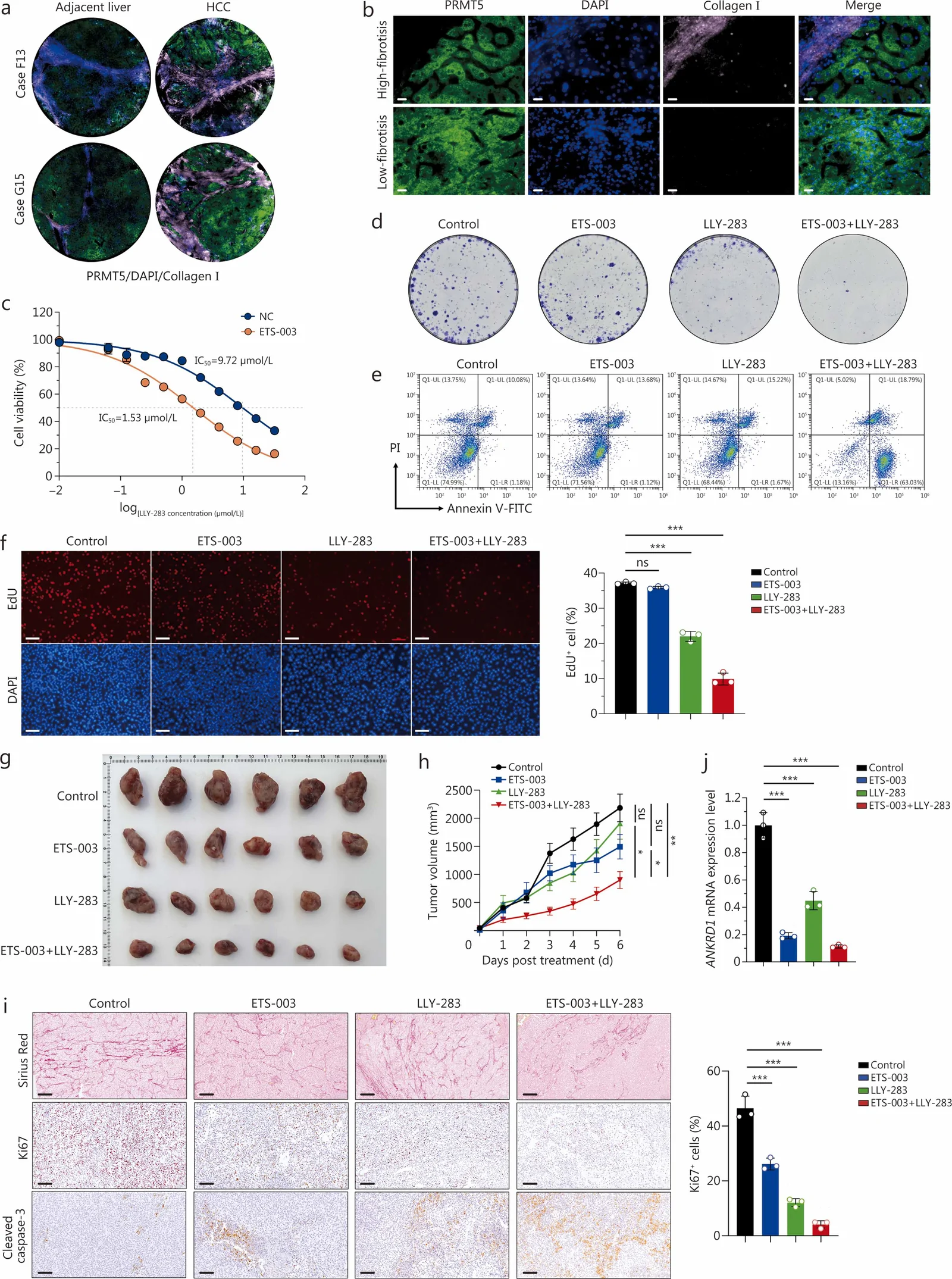
YAP/TAZ inhibitors increase the therapeutic efficacy of PRMT5 inhibitor in fibrosis-related HCC xenograft model. **a** Representative fluorescence images of PRMT5 (green), collagen I (pink), and nuclei (blue) in HCC specimens and paired adjacent liver tissues. **b** Representative fluorescence images of PRMT5 (green), collagen I (pink), and nuclei (blue) in HCC specimens. Scale bar= 50 μm. **c** Drug toxicity dose-response curves of LLY-283 in the presence or absence of ETS-003 in Mahlavu cells. **d** Representative images of foci formation assay of Mahlavu cells after treatment with ETS-003, LLY-283, or their combination. **e** Representative flow cytometry images of apoptosis assay of Mahlavu cells after treatment with ETS-003, LLY-283, or their combination. **f** Representative fluorescence images of EdU assay of Mahlavu cells after treatment with ETS-003, LLY-283, or their combination and bar charts showing the percentages of EdU^+^ cells (*n*=3). Scale bar=50 μm. *t*-test was performed vs the control group for all treatment groups. Dot-line plot showing images of resected tumour tissues (**g**) and tumour volume growth from mice with HCCLM3/LX-2 xenografts (**h**) after treatment with ETS-003, LLY-283, or their combination (*n*=6). Mice bearing HCCLM3/LX-2 xenografts were treated with a daily gavage of ETS-003 [50 mg/(kg·d)], an intraperitoneal injection of LLY-283 [20 mg/(kg·d)], or their combination. **i** Representative images of Sirius Red staining and IHC staining for Ki67 and cleaved caspase-3 expression in HCCLM3/LX-2 xenografts. Scale bar=100 μm. **j** Bar charts showing the results of the qPCR analysis of *ANKRD1* mRNA expression levels in HCCLM3/LX-2 xenografts (*n*=3). *t*-test was performed vs the control group for all treatment groups. The values represent the mean±SD. ^⁎^*P*<0.05, ^⁎⁎^*P*<0.01, ^⁎⁎⁎^*P*<0.001, ns non-significant. YAP. Yes1 associated transcriptional regulator; TAZ. Tafazzin; HCC. Hepatocellular carcinoma; PRMT5. Protein arginine methyltransferase 5; DAPI. 4’,6-diamidino-2-phenylindole; EdU. 5-ethynyl-2’-deoxyuridine; NC. Negative control; PI. Propidium iodide; FITC. Fluorescein isothiocyanate; ANKRD1. Ankyrin repeat domain 1; IC_50_. Half maximal inhibitory concentration; SD. Standard deviation.

In the former part of this study, we demonstrated that stiffness-induced ANKRD1 expression was driven mainly by the Hippo pathway, which could be potently suppressed by small-molecule inhibitors of YAP/TAZ-mediated transcription. Therefore, we investigated whether YAP/TAZ-TEAD inhibitors could sensitize Mahlavu cells to the PRMT5 inhibitor LLY-283. Therefore, we first performed a cytotoxicity assay with LLY-283 in the presence and absence of ETS-003, a YAP/TAZ-TEAD inhibitor, and found that the IC_50_ of LLY-283 decreased in the presence of ETS-003 in Mahlavu cells (Fig. 7**c**; Additional file 1: Fig. S6a**, b**). Similarly, the results of the foci formation assay, apoptosis assay, and EdU assay demonstrated that, compared with ETS-003 or LLY-283 alone, the combination of ETS-003 and LLY-283 markedly reduced malignant clonogenicity and proliferation, as well as induced cell apoptosis *in vitro* (Fig. 7**d-f;** Additional file 1: Fig. S6c). These results indicated that ETS-003 and LLY-283 exerted synergistic anti-HCC effects *in vitro*.

Furthermore, a fibrosis-related HCC xenograft model was established to better simulate HCC patients with concomitant fibrosis via subcutaneous inoculation of a mixture of an HCC cell line (HCCLM3) and a hepatic stellate cell line (LX-2). The images and tumour volume curves revealed that combination therapy significantly suppressed tumour growth, whereas the effects of the monotherapies were relatively weaker (Fig. 7**g, h**). Specifically, Sirius Red staining of resected HCC xenograft tissues revealed obvious collagen deposition in the subcutaneous HCCLM3/LX-2 xenografts. Additionally, the lowest percentage of Ki67^+^ cells and the most extensive region of cleaved caspase-3-positive staining were detected in the tumours treated with the combination therapy, which was consistent with the trend in the tumour growth curve (Fig. 7**i**). Finally, qPCR analysis of the tumour tissues confirmed that *ANKRD1* mRNA expression was significantly suppressed in both groups receiving ETS-003 as monotherapy or combination therapy (Fig. 7**j**).

Taken together, these data demonstrate that YAP/TAZ inhibitors can increase the therapeutic efficacy of PRMT5 inhibitor in HCC, especially in a fibrosis-related HCC xenograft model.

## 4 Discussion

In this study, we demonstrated that stiffness promoted the malignant proliferation of HCC cells. We identified ANKRD1 as one of the oncogenic proteins induced by the fibrotic microenvironment via the Hippo-YAP/TAZ pathway. Further mechanistic studies revealed that ANKRD1 enhanced the cytoplasmic localization and enzymatic activity of PRMT5 through protein-protein interaction. *ANKRD1* silencing sensitized HCC cells to a PRMT5 inhibitor, and the combination of a YAP/TAZ inhibitor and a PRMT5 inhibitor exhibited synergistic antitumour activity in a fibrosis-associated HCC model (Fig. 8).

**Fig. 8:**
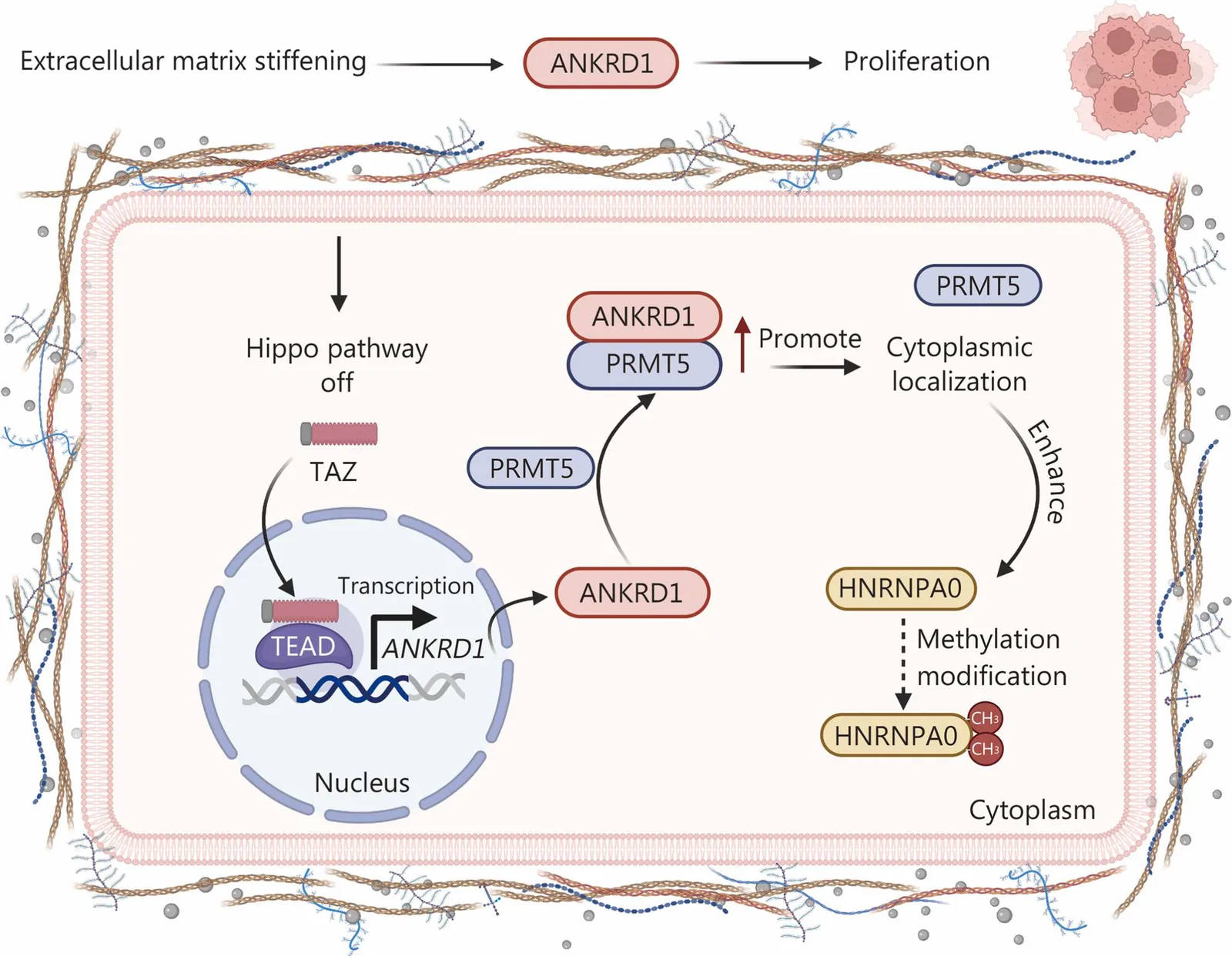
Mechanistic illustration of how matrix stiffness promotes the proliferation of HCC via the Hippo/ANKRD1/PRMT5 axis. ANKRD1. Ankyrin repeat domain 1; PRMT5. Protein arginine methyltransferase 5; HNRNPA0. Heterogeneous nuclear ribonucleoprotein A0.

Our work explored how the mechanobiological signalling pathway influences post-translational protein modifications from the perspective of the Hippo/ANKRD1/PRMT5 axis, and our preliminary results demonstrated that sDMA modification of HNRNPA0 could be increased by this axis. HNRNPA0 is an RNA-binding protein that regulates nuclear pre-mRNA splicing, nuclear export, and the cytoplasmic stability of mRNA by binding to AU-rich elements of mRNAs [49]. Post-translational modifications of HNRNPA0 are highly important for the dynamic regulation of its subcellular localization and biological functions. For example, a study on gastrointestinal epithelial tumours demonstrated that phosphorylation of HNRNPA0 increases its binding to RAB3 GTPase activating protein catalytic subunit 1 (*RAB3GAP1*) mRNA, thereby maintaining tumour mitotic events via RAB3GAP1-mediated stabilization of ZW10 interacting kinetochore protein (ZWINT1) [50]. The significance of sDMA modification of HNRNPA0 in HCC remains unclear. Our work found that silencing of *HNRNPA0* significantly suppressed the proliferation of HCC cells, whereas simultaneous silencing of both *ANKRD1* and *HNRNPA0* did not produce an additive inhibitory effect on HCC cell proliferation. Disordered RNA splicing may serve as a potential mechanism by which HNRNPA0 facilitates the proliferation of HCC cells, and further in-depth mechanistic investigations are still required to strengthen this point. Interestingly, our GSEA of the transcriptome of the TCGA-LIHC cohort revealed that RNA metabolism pathways were strongly affected by ANKRD1 expression. Given that our initial data revealed that the sDMA modification level of HNRNPA0 is positively regulated by ECM stiffness, it would be both biologically rational and scientifically compelling to further investigate whether ECM stiffness modulates RNA metabolism via the Hippo/ANKRD1/PRMT5 signalling axis.

In addition to RNA metabolism, PRMT5 plays a key role in epigenetic regulation through the modulation of histone methylation. Our study revealed that PRMT5 may act as a major downstream mediator but not necessarily the only effector of ANKRD1 induced by matrix stiffness. In the present study, as ANKRD1 was primarily localized in the cytoplasm, we did not explore its involvement in epigenetic regulatory events in the nucleus in depth. Epigenetic regulation is a key step in the regulation of gene expression, and multiple studies have reported that DNA methylation and microRNAs regulate the activation status of the Hippo pathway via epigenetic mechanisms [51], [52], [53]. However, the reverse regulatory direction, namely the regulation of epigenetic events by the Hippo pathway, is less frequently reported. This might be because epigenetic regulation often manifests as a long-term life regulatory process, whereas mechanobiology typically focuses on dynamic biomechanical regulatory processes. Nonetheless, overlaps exist between these two fields since biomechanical signal alterations may elicit enduring biological effects through epigenetic regulatory mechanisms during certain stages of embryonic development and pathological processes, such as malignant transformation [54]. In future studies, we plan to use the research system described herein to further investigate whether ECM stiffness exerts more extensive effects on histone methylation-based epigenomics.

In this study, several YAP inhibitors were used, some of which are currently in intensive clinical translation research for antitumour therapy (NCT04659096, NCT04665206, NCT05228015, and NCT04857372). Given the critical role of Hippo pathway dysregulation during disordered tissue proliferation, hyperactivated YAP/TAZ is a valid antitumour target, as it remains inactive in most tissues under homeostatic conditions [55], [56]. In basic oncology research, a comprehensive understanding of cellular signal transduction mechanisms enables the identification of more effective pharmaceutical targets or innovative combination therapies that leverage approved small-molecule drugs, thereby providing additional options for the systemic treatment of HCC in the future. Overall, this study provides a prospective theoretical basis for its application in the arms race against HCC.

## 5 Conclusions

ANKRD1 is a mechanically responsive protein that promotes the proliferation of HCC cells by regulating PRMT5 activity. The combination of YAP/TAZ inhibitors and a PRMT5 inhibitor is a potential therapeutic strategy for HCC patients with stiffened TME.

## Abbreviations

ΔC: C-terminal truncation
ΔM: Middle region truncation
ΔN: N-terminal truncation
Abcb4: ATP-binding cassette subfamily B member 4
ACTA2: Actin alpha 2, smooth muscle
ANKRD1: Ankyrin repeat domain protein 1
CAPZA2: Capping actin protein of muscle Z-line subunit alpha 2
Cas9: CRISPR-associated protein 9
CCK-8: Cell counting kit-8
CCN2: Cellular communication network factor 2
ChIP: Chromatin immunoprecipitation
Co-IP: Co-immunoprecipitation
COL1A1: Collagen type I alpha 1 chain
COL3A1: Collagen type III alpha 1 chain
COL6A1: Collagen type Ⅵ alpha 1 chain
CRISPR: Clustered Regularly Interspaced Short Palindromic Repeats
CTGF: Connective tissue growth factor
DAPI: 4’,6-diamidino-2-phenylindole
DBN1: Drebrin 1
ECM: Extracellular matrix
EdU: 5-ethynyl-2’-deoxyuridine
EPDR1: Ependymin related 1
FC: Fold change
FITC: Fluorescein isothiocyanate
FL: Full
FPKM: Fragments per kilobase of transcript per million mapped reads
GAPDH: Glyceraldehyde-3-phosphate dehydrogenase
GMT: Gene matrix transposed
GSA: Genome Sequence Archive
GSEA: Gene set enrichment analysis
GSVA: Gene set variation analysis
HA: Hemagglutinin
HCC: Hepatocellular carcinoma
HNRNPA0: Heterogeneous nuclear ribonucleoprotein A0
HNRNPC: Heterogeneous nuclear ribonucleoprotein C
HR: Hazard Ratio
HSPA8: Heat shock protein family A (Hsp70) member 8
IB: Immunoblotting
IC50: Half maximal inhibitory concentration
IF: Immunofluorescence
IGHV1–18: Immunoglobulin heavy variable 1–18
IHC: Immunohistochemistry
IP: Immunoprecipitation
KO: Knockout
LATS1/2: Large tumor suppressor kinase 1/2
LOX: Lysyl oxidase
MST1/2: Mammalian sterile 20-like kinase 1/2
MS: Mass spectrometry
Mut: Mutant
MYL6: Myosin light chain 6
NC: Negative control
OD: Optical density
PCBP2: Poly(rC) binding protein 2
PI: Propidium iodide
PRMT5: Protein arginine methyltransferase 5
RAB3GAP1: RAB3 GTPase activating protein catalytic subunit 1
RPL4: Ribosomal protein L4
RNA-seq: RNA sequencing
RT-qPCR: Real-time quantitative polymerase chain reaction
SD: Standard deviation
SE: Skipping exon
sDMA: Symmetric dimethylarginine
TAZ: Tafazzin
TCGA-LIHC: The Cancer Genome Atlas Liver Hepatocellular Carcinoma
TEMED: N,N,N’,N’-tetramethylethylenediamine
TIMP1: TIMP metallopeptidase inhibitor 1
TIMP2: TIMP metallopeptidase inhibitor 2
TME: Tumour microenvironment
TXN: Thioredoxin
WT: Wild type
YAP: Yes1 associated transcriptional regulator
ZWINT1: ZW10 interacting kinetochore protein

## Funding

This work was supported by the National Natural Science Foundation of China (82373888 to YTQ, 82372664 to JXC), the Non-profit Central Research Institute Fund of Chinese Academy of Medical Sciences (2023-PT320-02 to YTQ), the Joint Foundation of National Administration of Traditional Chinese Medicine & Zhejiang Province - Major Project (GZY-ZJ-KJ-24045 to JXC), and the Hangzhou Normal University Dengfeng Project “Clinical Medicine Revitalization Plan” Jiande Hospital Special Project (LCYXZXJH003 to JXC).

## Data Availability

The raw RNA-seq data for HCC cells (Mahlavu and HLE) cultured on stiff and soft substrates were deposited in the National Genomic Data Center under the Genome Sequence Archive (GSA)-human system with the accession number HRA013362 (https://ngdc.cncb.ac.cn/). All data that support the findings of this study are available from the corresponding authors upon reasonable request.

## Footnotes

Appendix A Supplementary data associated with this article can be found in the online version at doi:10.1016/j.mmr.2026.100075.
