# Supplementary material for: Matrix‑stiffness‑triggered mechanotransduction fuels hepatocellular carcinoma proliferation through Hippo‑orchestrated ANKRD1‑PRMT5 complex‑driven arginine methylome remodeling
Source: Mil Med Res. 2026 Oct 24;13(1):100075. doi: 10.1016/j.mmr.2026.100075 (PMC13720479; doi:10.1016/j.mmr.2026.100075)
Supplement: Supplementary file 1 [file mmc1.pdf]

Methods

Cell culture

The human hepatocellular carcinoma (HCC) cell lines Mahlavu, HLE, and SNU-886 were kindly gifted by Prof. Hui Kam Man (National Cancer Center Singapore). The human HCC cell line HCCLM3 and the human hepatic stellate cell line LX-2 were obtained from the China Center for Culture Collection. The mouse HCC cell line Hep-53.4 was purchased from the Meisen Chinese Tissue Culture Collection. Mahlavu, HLE, Hep-53.4, and LX-2 cells were maintained in Dulbecco's modified Eagle's medium (DMEM), while HCCLM3 and SNU-886 cells were cultured in Roswell Park Memorial Institute 1640 (RPMI 1640) medium. All culture media were supplemented with 10% foetal bovine serum (FBS) and 1% penicillinstreptomycin. Assessments of short tandem repeats and mycoplasmas were routinely performed.

Chemicals and plasmids

GNE-7883, a pan-TEA domain transcription factor (TEAD) inhibitor that disrupted the association between Yes1-associated transcriptional regulator/tafazzin (YAP/TAZ) and TEAD, was purchased from MedChemExpress (USA). ETS-003 and ETS-006, which are highly potent and orally available YAP(TAZ)/TEAD protein-protein interaction inhibitors, were provided by ETERN Therapeutics Co., Ltd. Small interfering RNA (siRNA) was synthesized by Beijing Tsingke Biotech Co., Ltd. CALN^{TM} RNAi *in vitro* (D-Nano Therapeutics, China) was used for siRNA transfection. The coding sequences of 3× Flag-tagged ankyrin repeat domain protein 1 (ANKRD1), hemagglutinin (HA)-tagged protein arginine methyltransferase 5 (PRMT5), and the ANKRD1 N-terminal/C-terminal/middle region truncation mutants were cloned and inserted into the pLVX-Puro vector. Virus packaging was performed in HEK-293T cells. For the dual-luciferase reporter assay, the wild type (WT) or mutant promoter region was cloned and inserted into the pGL3-Basic vector. Plasmids were constructed with the assistance of HZREPOBIO, China. Detailed information on the chemicals, oligonucleotides, and plasmids was listed in the **Additional file 1: Table S2**.

Sirius Red staining

Sirius Red staining was performed according to the manufacturer's protocol (Abcam, UK). Paraffin-embedded mouse and human HCC tissue sections were immersed in xylene for dewaxing, immersed in ethanol, and then washed 3 times with PBS. The sections were then immersed in Picro Sirius Red

solution for 1 h at room temperature. The tissue sections were rinsed twice with acetic acid solution, followed by rinsing with absolute alcohol to remove the Sirius Red solution. The sections were dehydrated twice in absolute alcohol and mounted with neutral resin.

Western blotting analysis

Western blotting was performed as previously described [1]. Cells were harvested and lysed in RIPA buffer (Beyotime, China) containing a cocktail of protease and phosphatase inhibitors on ice for 0.5 h with sonication to achieve complete lysis. The protein concentration was quantified using the Pierce™ Rapid Gold BCA Protein Assay Kit (Thermo Fisher Scientific, USA), and all the samples were diluted to 20 μg/10 μl. The protein samples were loaded onto an SDS-PAGE gel (GenScript, USA) and subjected to gel electrophoresis at a constant voltage until the dye reached the bottom. The proteins in the gels were transferred to PVDF membranes, which were subsequently incubated with 5% skim milk powder to reduce nonspecific antibody binding. The PVDF membrane was incubated with the primary antibody at 4 °C overnight. When the target protein and loading control differed significantly in molecular weight, the PVDF membrane from the same lane was cut and incubated separately with the respective antibodies. When the target proteins had similar molecular weights, the same amount of protein was loaded in the same order, and two parallel PVDF membranes were prepared for each target. The membranes were subsequently washed three times with 1× TBS containing 0.1% Tween-20 (TBST), followed by incubation with the secondary antibody at room temperature for 1 h. The membranes were then washed 3 times with TBST. ECL substrate was added to the band, and images were captured by the chemiluminescence system after a suitable exposure time.

Immunohistochemical staining (IHC)

IHC staining was performed as previously described [1]. Briefly, fresh tissue was immersed in formalin for 3 d, after which the paraffin-embedded tissue was cut into sections. After dewaxing and hydration, the sections were immersed in citric acid buffer or EDTA, and antigen retrieval was performed. The tissue was blocked with 5% bovine serum albumin (BSA) for 30 min, and the primary antibody was added for incubation at 4 °C overnight. After the sections were washed with PBS 3 times, the secondary antibody was added, and the sections were incubated at 37 °C for 0.5 h. The sections were then washed 3 times, after which diaminobenzidine was added for colour development. The sections were counterstained with haematoxylin and then mounted with neutral resin after dehydration.

Cell cycle analysis

Cell Cycle Staining Kit (MULTI SCIENCES, China) was used according to the manufacturer's protocol. The 5×10^5 cells were collected and washed with PBS, after which the supernatant was discarded. One millilitre of DNA staining solution and 10 μl of permeabilization solution were added to the cell pellet, which was vortexed for 5 s. The cells were then incubated at room temperature for 0.5 h. The samples were analysed using flow cytometry, and the results were analysed using ModFit LT (Verity Software House, USA).

Real-time quantitative polymerase chain reaction (RT-qPCR)

Total RNA was extracted using the AFTSpin Tissue/Cell Fast RNA Extraction Kit for Animal (ABclonal, China) according to the manufacturer's instructions. The quality and concentration of the total RNA were determined using a NanoDrop spectrophotometer. HiScript IV All-in-One Ultra RT SuperMix for qPCR (Vazyme, China) was used for reverse transcription. ChamQ Universal SYBR qPCR Master Mix (Vazyme, China) was used for RT-qPCR. The $2^{-\Delta\Delta CT}$ method was used for calculations on the basis of the cycle threshold. The primers used are listed in **Additional file 1: Table S2**. For tissue samples, lysis was added to thawed tissue and homogenized. The procedure was performed as previously described.

Cell counting kit-8 (CCK-8) assay

A cell suspension (100 μl) containing approximately 2000 cells was pipetted into a 96-well plate. Ten microliters of CCK-8 reagent were added to each well. The samples were incubated at 37 °C for 1 h, after which the absorbance at 450 nm was measured using a microplate reader (BioTek, USA).

Colony formation assay

Two thousand cells were seeded in 3 ml of complete medium in 6-well plates. The cells were cultured for several days until the cell clones reached approximately 0.5 mm. The clones were fixed with 4% paraformaldehyde (PFA) for 20 min and stained with crystal violet for 20 min.

5-Ethynyl-2'-deoxyuridine (EdU) proliferation assay

EdU proliferation assays were performed using a BeyoClick™ EdU Cell Proliferation Kit with Alexa Fluor 594 (Beyotime, China). Cells were seeded in 6-well plates and cultured overnight. EdU was then added at a final concentration of 10 μmol/L. The cells were cultured for another 2 h, after which the

supernatant was discarded. The cells were fixed with 4% PFA for 20 min and then washed with PBS 3 times. Next, 0.3% Triton X-100 was added, followed by incubation for 20 min and washing twice with PBS. Click reaction buffer (500 µl) was added according to the manufacturer's instructions, and the mixture was incubated for 0.5 h. DAPI was used to stain the nuclei. The percentage of EdU-positive cells was then calculated.

Immunoprecipitation (IP) and mass spectrometry (MS) analyses

Mahlavu, HLE, and HEK-293T cells were harvested and washed with pre-chilled PBS, and the cell pellet was lysed using IP lysis buffer (Beyotime, China) containing protease and phosphatase inhibitors. The lysate was placed on ice for 0.5 h and vortexed every 10 min. After centrifugation at 12000× *g* and 4 °C for 15 min, the supernatant was pipetted into new tubes. The protein concentration was determined using the BCA assay, and all protein samples were diluted to 1 µg/µl. Protein samples (1 ml) were prepared for co-immunoprecipitation (co-IP), and 10% of each sample was reserved for input. The protein samples were incubated with antibody-conjugated beads at 4 °C overnight. The protein-antibody-bead complexes were washed 3 times with IP wash buffer and boiled with 1× SDS loading buffer. The subsequent steps were similar to those used for western blotting.

IP and MS analyses were performed to identify the proteins that interact with ANKRD1. Flag-ANKRD1 was precipitated as previously described, and 500 µl of NH_4HCO_3 was used for elution. The samples were then reduced with 1 mmol/L dithiothreitol for 30 min at 65 °C. And 0.5 µg of trypsin was added, and the samples were digested overnight at 37 °C. An additional 0.5 µg of trypsin was added for 3 h of incubation at 37 °C. Indoleacetic acid (2.5 mmol/L) was added, and the samples were incubated at room temperature for 0.5 h in the dark. The pH was adjusted to 2–3 using formic acid, and the supernatant was collected. The peptide was dried. Desalination was performed using a SOLA™ SPE column (Thermo Fisher Scientific, USA). Two mobile phases were prepared: mobile phase A (0.1% formic acid in water) and mobile phase B (0.1% formic acid in acetonitrile). The enzymatically digested peptides were dissolved in mobile phase A and loaded onto an RP-C18 trap column (15 cm×100 µm, 1.9 µm). Gradient elution was performed as follows: 0–7.5 min, 6%–28% mobile phase B; 7.5–9 min, 28%–80% mobile phase B; and 9–10 min, 80% mobile phase B at flow rates of 700, 400, 700, and 700 nl/min. The peptides were then injected into a capillary ion source for electrospray ionization, followed by data acquisition using a timsTOF HT mass spectrometer (Bruker Daltonics, Germany) in data-independent acquisition with parallel accumulation serial fragmentation

(dia-PASEF) mode. The ion source voltage was set to 1.6 kV, and the drying temperature was set to 180 °C. The full scan range of the primary mass spectrum (MS1) was set at 300–1500 *m/z*. After the first scan (MS1) was collected, 20 PASEF mode scans were performed. For the secondary mass spectrum (MS2) scans, the range was set to 400–850 *m/z* with a window size of 7 *m/z* per segment. MS/MS data were analysed by PASER (Bruker) using UniProt-Homo sapiens-9606-2024.2.1.fasta. Trypsin was used as the cleavage enzyme, and one missing cleavage was allowed. Cysteine (C) carbamidomethylation was set as a fixed modification, while variable modifications included methionine oxidation and acetylation of the protein N-terminus. The false discovery rate (FDR) for protein, peptide, and peptide-spectrum match (PSM) identification was set to 1%.

Immunofluorescence (IF) staining

Methanol was pre-chilled at –20 °C for 30 min, after which the Mahlavu were fixed and permeabilized simultaneously with the pre-chilled methanol at –20 °C for 15 min. After being washed twice with PBS, antigen blocking was performed with 5% BSA at room temperature for 1 h. The primary antibody was added to the cells after suitable dilution for incubation at 4 °C overnight. The samples were then washed three times and incubated with a secondary antibody at 37 °C for 30 min. Anti-fade mounting medium containing DAPI (YEASEN, China) was used to stain the nuclei and immobilize the coverslips. Images were obtained using a confocal laser scanning microscope (Olympus FV3000, Japan).

Dual-luciferase reporter assay

The TAZ-binding promoter region of ANKRD1, validated by ChIP-qPCR, was cloned and inserted into the pGL3-Basic vector. Then, 0.2 μg of pRL-SV40 and 2 μg of WT or mutant pGL3-Basic-promoter plasmids were co-transfected into Mahlavu cells for culture on soft and stiff substrates. After being cultured for 24 h, the cells were harvested and lysed. The lysate (20 μl) was pipetted into 100 μl of firefly luciferase working solution, and the luminescence was measured. Renilla luciferase containing a quenching agent (100 μl) was added, and the luminescence was measured again. The relative luciferase activity was calculated as the ratio of firefly luciferase luminescence to Renilla luciferase luminescence.

RNA sequencing

RNA sequencing was performed by Shanghai OE Biotech Co., Ltd. Briefly, total RNA was extracted,

and rRNA was removed. The RNA was fragmented and reverse-transcribed into cDNA. The library was constructed using TruSeq Stranded Total RNA Ribo-Zero H/M/R Gold, following the manufacturer's protocol. RNA sequencing was performed using HiSeq^{TM} 2500, and paired-end reads were generated as FASTQ files. For data analysis, Trimmomatic [2] was used to filter out adaptors and low-quality bases. Alignment of the clean reads against the human genome was performed using Hisat2 [3]. The reads were assembled using StringTie [4]. Gene quantification was performed using HTSeq-count [5], and a count matrix was generated. DESeq2 [6] was used to normalize counts and calculate the fragments per kilobase of transcript per million mapped reads (FPKM) and perform differential expression analysis. RNA splicing was analyzed by rMATS (v4.1.2) with the parameters '-*t* paired-libType-readLength 150 -tstat 5-cstat 0.0001' [7].

Chromatin immunoprecipitation (ChIP)

ChIP was performed using the Pierce™ Magnetic ChIP Kit (Thermo Fisher Scientific, USA) as described previously [8]. The cells were fixed and crosslinked with 1% formaldehyde at room temperature for 10 min, followed by quenching with 1× glycine. The nuclei were extracted, digested with micrococcal nuclease, and sonicated under suitable conditions to obtain fragmented chromatin with a length of 200–1000 bp. The DNA fragments were incubated with primary antibody at 4 °C overnight with shaking and then incubated with protein A/G beads for 2 h with shaking the next day. After the samples were washed with wash buffer 1 and wash buffer 2, the antigen-antibody-chromatin complex was eluted at 65 °C for 30 min. The protein in the samples was eliminated by incubation with proteinase K, and decrosslinking was performed simultaneously at 65 °C for 1.5 h. Chromatin was extracted using a column-based method.

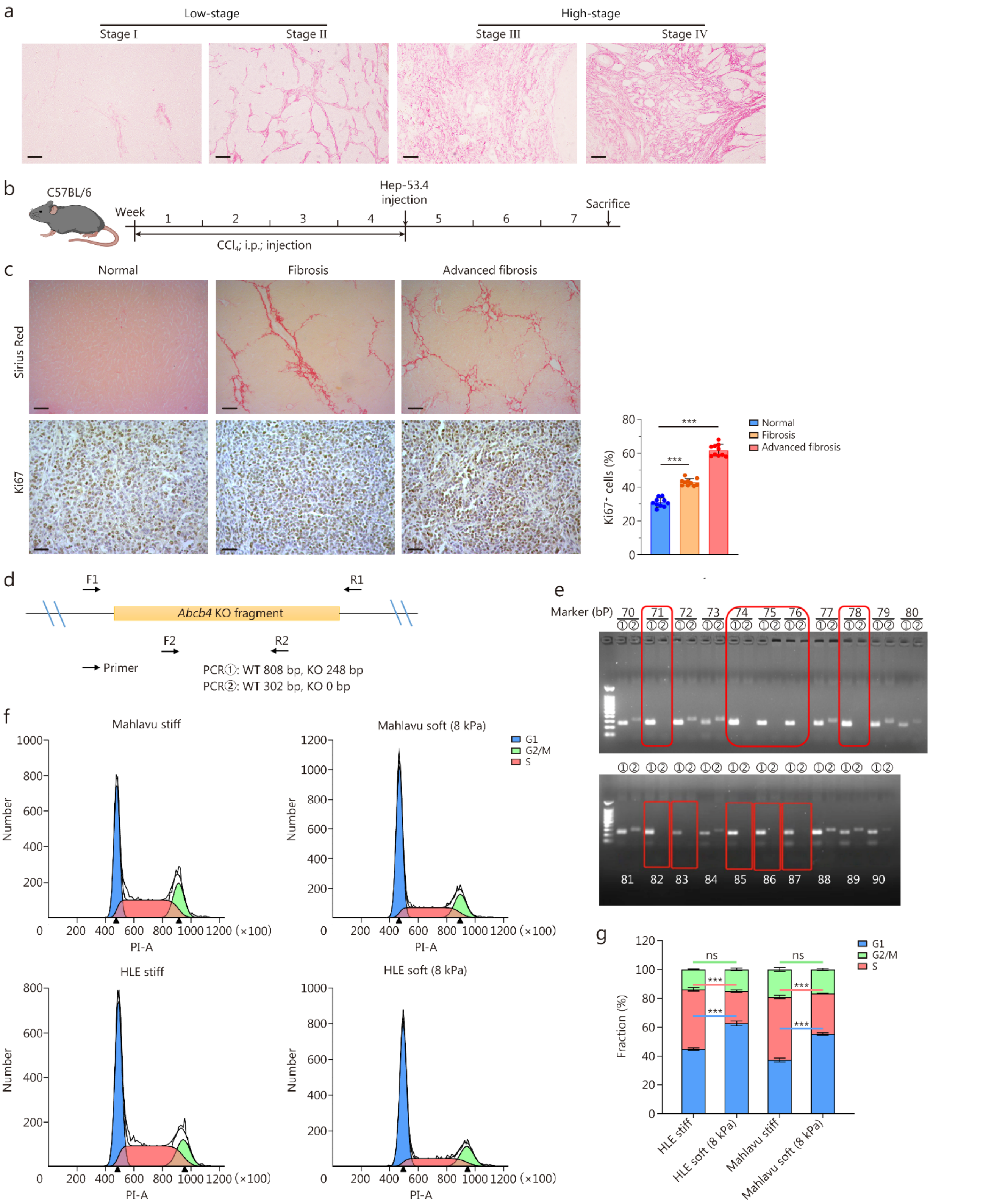

Fig. S1 Fibrotic TME promotes the malignant progression of HCC. **a** Sirius Red staining of HCC tissues at different stages. The HCC stage information was derived from the clinical pathological reports. Scale bar=100 μm. **b** Illustration of the orthotopic HCC model with the murine HCC cell line Hep-53.4 in mice with normal, fibrotic, or advanced fibrosis livers. **c** Sirius red staining and Ki67 images and the quantification of normal, fibrotic, and advanced fibrosis livers in the tumour and

adjacent liver tissue. Scale bar=100 μm. Illustration of the primer design (**d**) and gel electrophoresis (**e**) for the validation of *Abcb4*$^{-/-}$ mice. The red frame indicates *Abcb4*$^{-/-}$ mice. Mice genotyping gel for *Abcb4*$^{-/-}$ (primer pair ①: WT 808 bp, KO 248 bp; primer pair ②: WT 302 bp, KO 0 bp). The band is the PCR product for the corresponding primers. **f** Cell cycle analysis of Mahlavu and HLE cells cultured on stiff or soft substrates. **g** Stacked bar chart of the cell cycle analysis results. The values represent the mean±SD. $^{***}P<0.001$, ns non-significant. Abcb4. ATP-binding cassette subfamily B member 4; WT. Wild type; KO. Knockout; PI. Propidium iodide; TME. Tumour microenvironment; HCC. Hepatocellular carcinoma; SD. Standard deviation

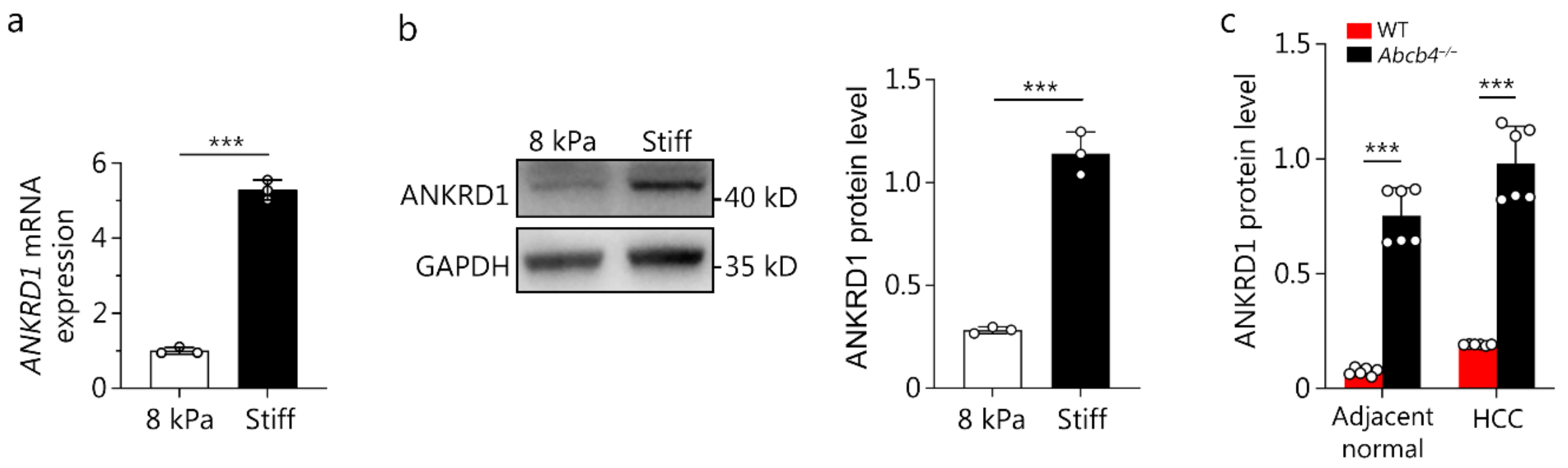

Fig. S2 Fibrotic TME induces ANKRD1 expression in HCC. **a** qPCR results showing *ANKRD1* expression levels in HLE cells cultured on stiff and soft substrates (n=3). t-test was performed using "8 kPa" as control. **b** Western blotting of ANKRD1 cultured on stiff and soft substrates (8 kPa). **c** Relative quantification of ANKRD1 protein level in HCC and adjacent normal liver from *Abcb4*$^{-/-}$ and WT mice. The values represent the mean±SD. $^{***}P<0.001$. ANKRD1. Ankyrin repeat domain 1; GAPDH. Glyceraldehyde-3-phosphate dehydrogenase; Abcb4. ATP-binding cassette subfamily B member 4; HCC. Hepatocellular carcinoma; TME. Tumour microenvironment; SD. Standard deviation

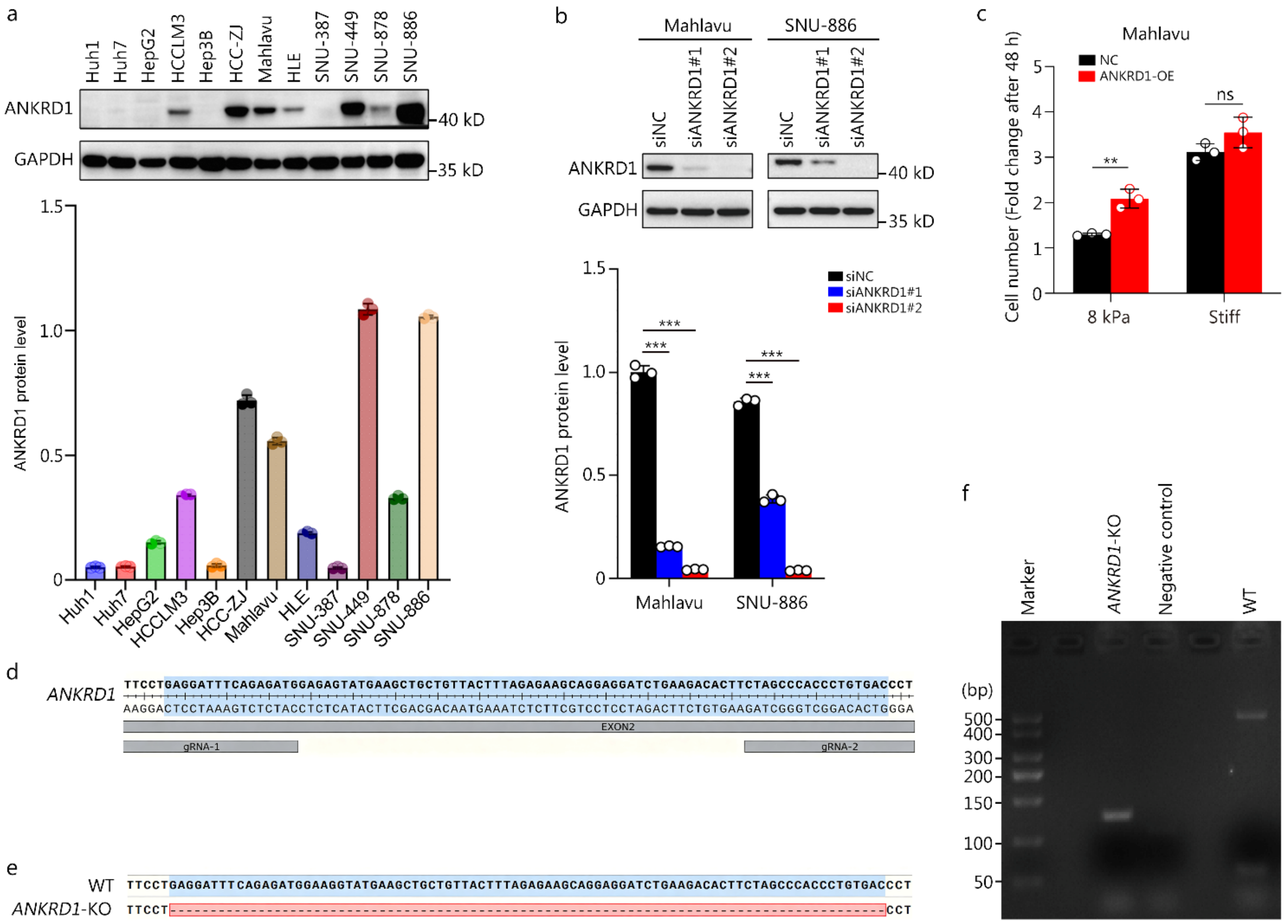

Fig. S3 ANKRD1 promotes the malignant progression of HCC. **a** Western blotting analysis of ANKRD1 expression in multiple HCC cell lines. **b** Western blotting analysis of ANKRD1 expression when Mahlavu and SNU-886 cells transfected with siANKRD1#1 and siANKRD1#2. **c** Grouped bar chart showing the fold change in cell number after ANKRD1 was overexpressed in Mahlavu cells on stiff and soft substrates (8 kPa) (*n*=3). **d** Illustration of the sgRNA location for CRISPR-Cas9. Sanger sequencing (**e**) and gel electrophoresis (**f**) results to confirm the knockout of *ANKRD1*. The values represent the mean±SD. $^{**}P<0.01$, ns non-significant. ANKRD1. Ankyrin repeat domain 1; GAPDH. Glyceraldehyde-3-phosphate dehydrogenase; HCC. Hepatocellular carcinoma; NC. Negative control; OE. Overexpression; WT. Wild type; KO. Knockout; SD. Standard deviation

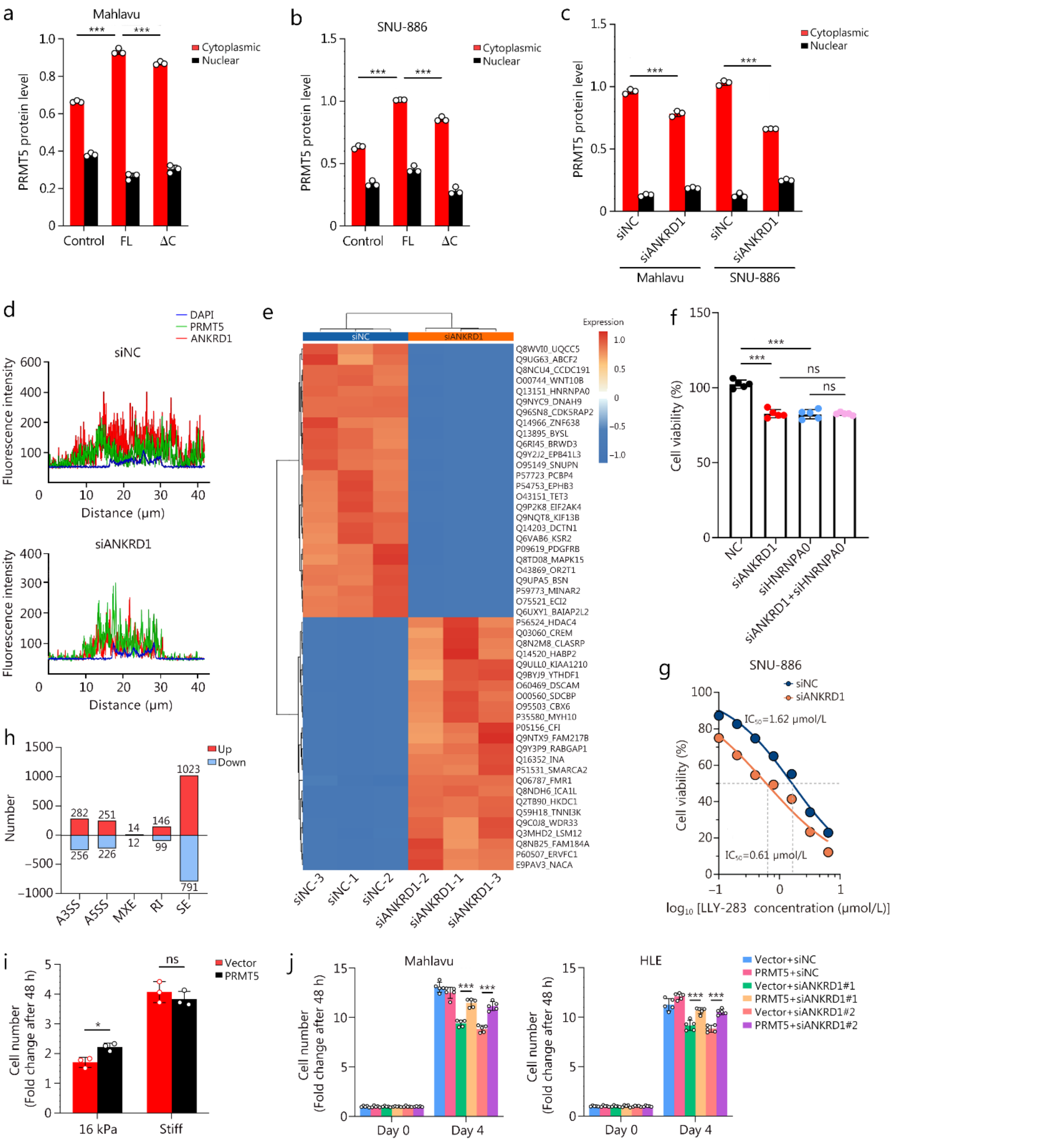

Fig. S4 ANKRD1 promotes the methyltransferase activity of PRMT5 in the cytoplasm. Relative quantification of cytoplasmic and nuclear PRMT5 transfected with control, full-length, or C-terminal truncation of ANKRD1 in Mahlavu (**a**) and SNU-886 (**b**). **c** Relative quantification of cytoplasmic and nuclear PRMT5 transfected with NC or siANKRD1. **d** Fluorescence intensity profile showing weaker ANKRD1-PRMT5 co-localization after *ANKRD1* knockdown. **e** Heatmap illustrating the differential expression profiles of sDMA-enriched proteins after ANKRD1 knockdown according to PRO-DIA proteomics. **f** The cell proliferation assay for the knockdown of *ANKRD1* or *HNRNPA0* and their combination. **g** Drug toxicity dose–response curves of LLY-283 in the presence or absence of siRNA

against ANKRD1 in SNU-886 cells cultured on stiff substrates. **h** The number of differential splicing events for ANKRD1 knockout. A3SS (Alternative 3’ Splice Site), A5SS (Alternative 5’ Splice Site), MXE (Mutually Exclusive Exons), RI (Retention Intron), and SE (Skipping Exon). **i** Grouped bar chart showing the fold change in the number of Mahlavu cells overexpressing PRMT5 cultured on either soft or stiff substrates (n=3). **j** Relative cell numbers of *ANKRD1* knockdown, PRMT5 overexpression, and their combination in Mahlavu and HLE cells cultured on stiff substrates. The values represent the mean±SD. $^{*}P<0.05$, $^{***}P<0.001$, ns non-significant. PRMT5. Protein arginine methyltransferase 5; FL. Full; ΔC. C-terminal truncation; NC. Negative control; ANKRD1. Ankyrin repeat domain 1; HNRNPA0. Heterogeneous nuclear ribonucleoprotein A0; A3SS. Alternative 3’ Splice Site; A5SS. Alternative 5’ Splice Site; MXE. Mutually Exclusive Exons; RI. Retention Intron; SE. Skipping Exon; IC50. Half maximal inhibitory concentration; sDMA. Symmetric dimethylarginine; SD. Standard deviation

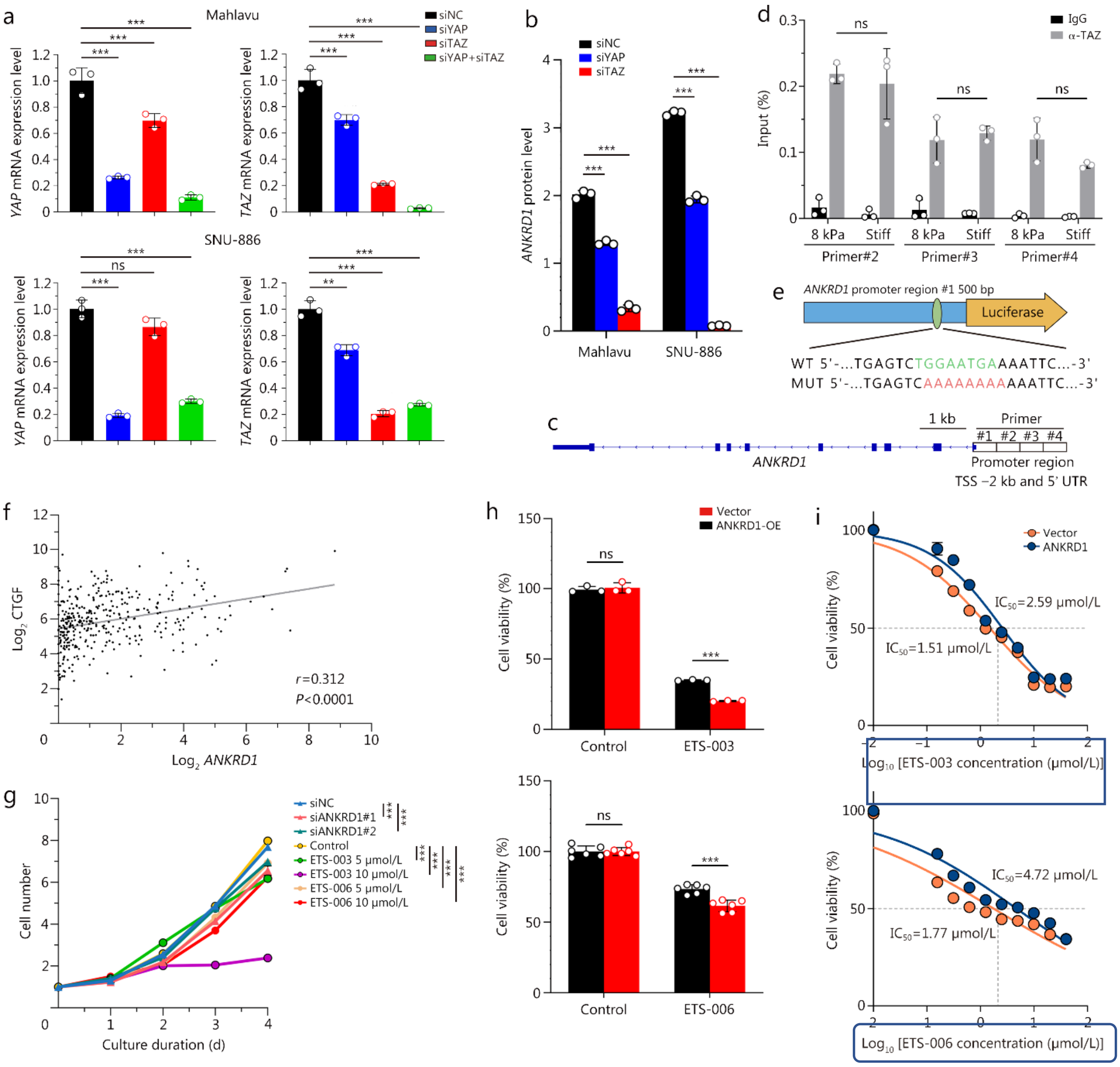

Fig. S5 Stiffness-induced ANKRD1 expression is mediated by the Hippo pathway in HCC. **a** *YAP* and *TAZ* mRNA expression in Mahlavu and SNU-886 cells transfected with siYAP, siTAZ, or their combination. **b** Relative quantification of ANKRD1 protein level in Mahlavu and SNU-886 cells transfected with siNC, siYAP, or siTAZ. **c** Illustration of primers targeting the *ANKRD1* promoter for ChIP-qPCR. **d** ChIP-qPCR results with primers #2, #3, and #4 in Mahlavu cells cultured on soft or stiff substrates. **e** Illustration of the design of the WT and mutant (MUT) promoters for the luciferase reporter assay. **f** Correlation between the expression of ANKRD1 and CTGF in the TCGA-LIHC cohort. **g** The cell growth curves treated with siANKRD1 and Hippo pathway inhibitors ETS-003 and ETS-006. Comparisons were performed between siANKRD1 and siNC, as well as the control and treatment of ETS-003 or ETS-006. **h** The relative cell viability of ANKRD1-overexpressing Mahlavu treated with the Hippo pathway inhibitors ETS-003 and ETS-006. **i** Dose-response curves of

ANKRD1-overexpressing HLE treated with the Hippo pathway inhibitors ETS-003 and ETS-006. The values represent the mean±SD. $^{**}P<0.01$, $^{***}P<0.001$, ns non-significant. YAP. Yes1 associated transcriptional regulator; TAZ. Tafazzin; NC. Negative control; ANKRD1. Ankyrin repeat domain 1; WT. Wild type; MUT. Mutant; TSS. Transcription start sites; UTR. Untranslated region; CTGF. Connective tissue growth factor; OE. Overexpression; IC50. Half maximal inhibitory concentration; ChIP. Chromatin immunoprecipitation; TCGA-LIHC. The Cancer Genome Atlas Liver Hepatocellular Carcinoma; SD. Standard deviation

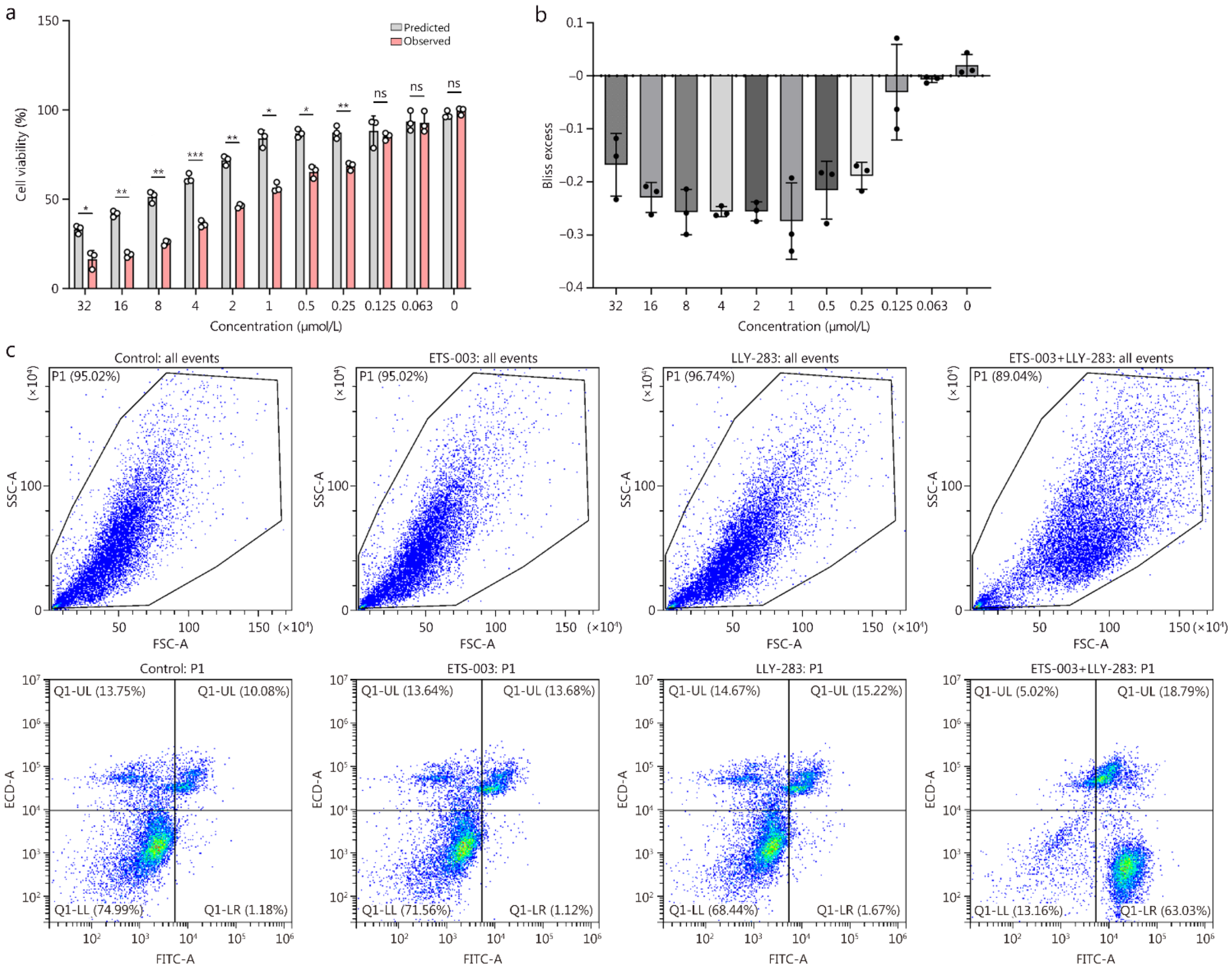

Fig. S6 YAP/TAZ inhibitors increase the therapeutic efficacy of PRMT5 inhibitor in fibrosis-related HCC xenograft model. **a** The predicted and observed cell viability for the combination of ETS-003 and LLY-283. **b** Histogram showing the Bliss excess that was calculated by subtracting the predicted cell viability from the observed cell viability. **c** Gating strategy of the flow cytometry for apoptosis assay in Fig. 7e. The values represent the mean±SD. $^{*}P$<0.05, $^{**}P$<0.01, $^{***}P$<0.001, ns non-significnat. YAP. Yes1 associated transcriptional regulator; TAZ. Tafazzin; PRMT5. Protein arginine methyltransferase 5; HCC. Hepatocellular carcinoma; SSC-A. Side scatter area; FSC-A. Forward scatter area; ECD-A. Energy-coupled dye area; FITC-A. Fluorescein isothiocyanate area; SD. Standard deviation

Table S1 Genes included in the HP_HEPATIC_FIBROSIS gene-set

**Genes**									
ASL	*MST1*	*INSR*	*SCYL1*	*ATP6AP1*	*RBCK1*	*PYGL*	*MET*	*LIPA*	*SLC25A13*
ABCD3	*CP*	*ACADVL*	*CLDN1*	*CTNNB1*	*TALDO1*	*ABCB4*	*MTTP*	*SLC51A*	*GPD1*
IFT27	*DGUOK*	*PHKG2*	*BCS1L*	*PTRH2*	*AGL*	*NOTCH1*	*MED12*	*DPM1*	*NGLY1*
NHP2	*MPI*	*TNPO3*	*PHKB*	*DOCK6*	*RBPJ*	*TXNDC15*	*TCTN3*	*ASAH1*	*DNASE2*
PNPLA6	*UNC45A*	*AP1S1*	*NOP10*	*MKKS*	*PHKA2*	*KIF3B*	*ANKS6*	*GLIS3*	*ARHGAP31*
SEMA4D	*PLIN1*	*PMM2*	*USP53*	*SCAPER*	*BBS10*	*WDR35*	*BBS9*	*NEK1*	*LZTFL1*
TTC8	*B9D1*	*NEK8*	*PRIM1*	*IFT74*	*TRIM32*	*INVS*	*ALG9*	*MKS1*	*BBIP1*
TMEM237	*IQCB1*	*CEP290*	*IFT140*	*EOGT*	*FADD*	*CYP7B1*	*HBB*	*DCDC2*	*KIF12*
DLL4	*TCF4*	*IRF5*	*CSPP1*	*INPP5E*	*TULP3*	*IFT172*	*BBS2*	*PEX1*	*TCTN1*
ALMS1	*WDR19*	*BBS4*	*UBR1*	*RINT1*	*TRAF3IP1*	*IFT122*	*SDCCAG8*	*OFD1*	*CEP164*
RNU7-1	*MMEL1*	*IL12A*	*POU2AF1*	*SPIB*	*TERT*	*PKHD1*	*GPR35*	*DZIP1L*	*TNFSF15*
DYNC2H1	*TMEM231*	*RPGRIP1*	*NPHP1*	*BBS1*	*CEP19*	*ARL6*	*BBS12*	*HAMP*	*SLC51B*
IL12RB1	*BBS5*	*WDPCP*	*TMEM67*	*RPGRIP1L*	*TTC26*	*NPHP4*	*CCDC28B*	*BBS7*	*IFT80*
SCLT1	*NPHP3*	*TMEM216*	*B9D2*	*TMEM107*	*CC2D2A*	*TCTN2*			

ASL. Argininosuccinate lyase; MST1. Macrophage stimulating 1; INSR. Insulin receptor; SCYL1. SCY1 like pseudokinase 1; ATP6AP1. ATPase H^+ transporting accessory protein 1; RBCK1. RANBP2-type and C3HC4-type zinc finger containing 1; PYGL. Glycogen phosphorylase L; MET. MET proto-oncogene, receptor tyrosine kinase; LIPA. Lipase A, lysosomal acid type; SLC25A13. Solute carrier family 25 member 13; ABCD3. ATP binding cassette subfamily D member 3; CP. Ceruloplasmin; ACADVL. Acyl-CoA dehydrogenase very long chain; CLDN1. Claudin 1; CTNNB1. Catenin beta 1; TALDO1. Transaldolase 1; ABCB4. ATP binding cassette subfamily B member 4; MTTP. Microsomal triglyceride transfer protein; SLC51A. Solute carrier family 51 subunit alpha; GPD1. Glycerol-3-phosphate dehydrogenase 1; IFT27. Intraflagellar transport 27; DGUOK. Deoxyguanosine kinase; PHKG2. Phosphorylase kinase gamma 2; BCS1L. BCS1 homolog, ubiquinol-cytochrome c reductase complex chaperone; PTRH2.

Peptidyl-tRNA hydrolase 2; AGL. Amylo-1,6-glucosidase, 4-alpha-glucanotransferase; NOTCH1. Notch receptor 1; MED12. Mediator complex subunit 12; DPM1. Dolichol-phosphate mannosyltransferase subunit 1; NGLY1. N-glycanase 1; NHP2. NHP2 ribonucleoprotein; MPI. Mannose phosphate isomerase; TNPO3. Transportin 3; PHKB. Phosphorylase kinase beta; DOCK6. Dedicator of cytokinesis 6; RBPJ. Recombination signal binding protein for immunoglobulin kappa J region; TXNDC15. Thioredoxin domain containing 15; TCTN3. Tectonic family member 3; ASAH1. N-acylsphingosine amidohydrolase 1; DNASE2. Deoxyribonuclease II, lysosomal; PNPLA6. Patatin like phospholipase domain containing 6; UNC45A. Unc-45 myosin chaperone A; AP1S1. Adaptor related protein complex 1 subunit sigma 1; NOP10. NOP10 ribonucleoprotein; MKKS. McKusick-Kaufman syndrome; PHKA2. Phosphorylase kinase alpha 2; KIF3B. Kinesin family member 3B; ANKS6. Ankyrin repeat and SAM domain containing 6; GLIS3. GLIS family zinc finger 3; ARHGAP31. Rho GTPase activating protein 31; SEMA4D. Semaphorin 4D; PLIN1. Perilipin 1; PMM2. Phosphomannomutase 2; USP53. Ubiquitin specific peptidase 53; SCAPER. S-phase cyclin A associated protein in the ER; BBS10. Bardet-Biedl syndrome 10; WDR35. WD repeat domain 35; BBS9. Bardet-Biedl syndrome 9; NEK1. NIMA related kinase 1; LZTFL1. Leucine zipper transcription factor like 1; TTC8. Tetratricopeptide repeat domain 8; B9D1. B9 domain containing 1; NEK8. NIMA related kinase 8; PRIM1. Primase subunit 1; IFT74. Intraflagellar transport 74; TRIM32. Tripartite motif containing 32; INVS. Inversin; ALG9. Alpha-1,2-mannosyltransferase; MKS1. Meckel syndrome type 1; BBIP1. BBSome interacting protein 1; TMEM237. Transmembrane protein 237; IQCB1. IQ motif and B1 domain containing 1; CEP290. Centrosomal protein 290; IFT140. Intraflagellar transport 140; EOGT. EGF domain specific O-linked N-acetylglucosamine transferase; FADD. Fas associated via death domain; CYP7B1. Cytochrome P450 family 7 subfamily B member 1; HBB. Hemoglobin subunit beta; DCDC2. Doublecortin domain containing 2; KIF12. Kinesin family member 12; DLL4. Delta like canonical Notch ligand 4; TCF4. Transcription factor 4; IRF5. Interferon regulatory factor 5; CSPP1. Centrosome and spindle pole associated protein 1; INPP5E. Inositol polyphosphate-5-phosphatase E; TULP3. Tubby like protein 3; IFT172. Intraflagellar transport 172; BBS2. Bardet-Biedl syndrome 2; PEX1. Peroxisomal biogenesis factor 1; TCTN1. Tectonic family member 1; ALMS1. ALMS1 centrosome and basal body associated protein; WDR19. WD repeat domain 19; BBS4. Bardet-Biedl syndrome 4; UBR1. Ubiquitin protein ligase E3 component n-recognin 1; RINT1. RAD50 interactor 1; TRAF3IP1. TRAF3 interacting protein 1; IFT122. Intraflagellar transport 122; SDCCAG8. Serologically defined colon cancer antigen 8; OFD1. Oral-facial-digital syndrome 1; CEP164. Centrosomal protein 164; RNU7-1. RNA, U7 small nuclear 1; MMEL1. Membrane metalloendopeptidase like 1; IL12A. Interleukin 12A; POU2AF1. POU class 2 homeobox associating factor 1; SPIB. Spi-B transcription factor; TERT. Telomerase reverse transcriptase; PKHD1. Polycystic kidney and hepatic disease 1; GPR35. G protein-coupled receptor 35; DZIP1L. DAZ interacting zinc finger protein 1 like; TNFSF15. TNF superfamily member 15; DYNC2H1. Dynein cytoplasmic 2 heavy chain 1; TMEM231. Transmembrane protein 231; RPGRIP1. RPGR interacting protein 1; NPHP1. Nephronophthisis 1; BBS1. Bardet-Biedl syndrome 1; CEP19. Centrosomal protein 19; ARL6. ADP ribosylation factor like GTPase 6; BBS12. Bardet-Biedl syndrome 12; HAMP. Hepcidin antimicrobial peptide; SLC51B. Solute carrier family 51 subunit beta; IL12RB1. Interleukin 12 receptor subunit beta 1; BBS5. Bardet-Biedl syndrome 5; WDPCP. WD repeat containing planar cell polarity effector; TMEM67. Transmembrane protein 67; RPGRIP1L. RPGRIP1 like; TTC26. Tetratricopeptide repeat domain 26; NPHP4. Nephronophthisis 4; CCDC28B. Coiled-coil domain containing 28B; BBS7. Bardet-Biedl syndrome 7; IFT80. Intraflagellar transport 80; SCLT1. Sodium channel and clathrin linker 1; NPHP3. Nephronophthisis 3; TMEM216. Transmembrane protein 216; B9D2. B9 domain containing 2; TMEM107. Transmembrane protein 107; CC2D2A. Coiled-coil and C2 domain containing 2A; TCTN2. Tectonic family member 2

Table S2 Reagent and resources

**Reagent and resource**	**Source**		**Cat#**
Antibodies			
Rabbit polyclonal anti-Ki67	Abcam	1:200 for IHC	ab15580
Mouse monoclonal anti-ANKRD1	Proteintech	1:5000 for WB	67775-1-Ig
Mouse monoclonal anti-GAPDH	Abclonal	1:200000 for WB	AC033
Rabbit polyclonal anti-ANKRD1	Absin	1:100 for IHC	abs147309
Rabbit monoclonal anti-PRMT5	Cell Signaling Technology	1:1000 for WB,<br>1:50 for IP, 1:200 for IF	79998
Mouse monoclonal anti-Flag	Sigma-Aldrich	1:1000 for WB	F1804
Rabbit monoclonal anti-β-actin	Abclonal	1:200000 for WB	AC026
Rabbit monoclonal anti-HA	Cell Signaling Technology	1:1000 for WB	3724
Rabbit polyclonal anti-Lamin B1	Abclonal	1:2000 for WB	A1910
Rabbit monoclonal anti-α-tubulin	Abclonal	1:20000 for WB	A6830
Mouse monoclonal anti-β-tubulin	Abclonal	1:5000 for WB	AC021
Rabbit monoclonal anti-Symmetric<br>Di-methyl arginine motif	Cell Signaling Technology	1:1000 for WB	13222
Rabbit polyclonal anti-HNRNPA0	Cell Signaling Technology	1:1000 for WB,<br>1:100 for IP	4046
Rabbit monoclonal anti-YAP	Cell Signaling Technology	1:1000 for WB	14074
Rabbit monoclonal anti-TAZ	Cell Signaling Technology	1:1000 for WB	83669
Rabbit polyclonal anti-TAZ	Sigma-Aldrich	1:200 for IHC	HPA007415
Rabbit polyclonal anti-collagen I	Proteintech	1:400 for IF	14695-1-AP
Rabbit polyclonal anti-cleaved caspase-3	Cell Signaling Technology	1:400 for IHC	9661

Reagent and resource	Source		Cat#
HRP-conjugated goat anti-rabbit IgG(H+L)	Proteintech	1:5000 for WB	SA00001-2
HRP-conjugated goat anti-mouse IgG(H+L)	Proteintech	1:5000 for WB	SA00001-1
Biological samples			
Surgical specimens from HCC patients	This paper		N/A
Chemicals, peptides, and recombinant proteins			
CCl_4	MedChemExpress		HY-Y0298
D-luciferin potassium	Beyotime		ST196
DNA/siRNA transfection reagent	JetPRIME		101000001
GNE-7883	MedChemExpress		HY-147214
ETS-003	ETERN Therapeutics Co., Ltd.		
ETS-006	ETERN Therapeutics Co., Ltd.		
Sulfo-SANPAH	Sigma-Aldrich		803332
Critical commercial assays			
Cell counting kit-8	MedChemExpress		HY-K0301
Picro Sirius Red stain kit	Abcam		ab150681
EdU	Beyotime		ST067
Pierce™ magnetic ChIP kit	Thermo Scientific		26157
Annexin V-FITC/PI apoptosis kit	Multi Sciences		AP101
Cell cycle staining kit	Multi Sciences		CCS012
HiScript IV all-in-One Ultra RT SuperMix for qPCR	Vazyme		R433-01
ChamQ universal SYBR qPCR master mix	Vazyme		Q711-02
Double-luciferase reporter assay kit	TransGen		FR201

Reagent and resource	Source	Cat#
Nuclear and cytoplasmic protein extraction kit	Beyotime	P0028
Pierce^{TM} rapid gold BCA protein assay kit	Thermo Scientific	A53225
Epigenase PRMT methyltransferase (Type II-specific) activity/inhibition Assay kit (colorimetric)	EpigenTek	P-3088
Deposited data		
RNA-seq (Mahlavu and HLE on soft and stiff cultured dish)	This paper	
RNA-seq (HCC samples)	TCGA-LIHC	
Immunoprecipitation-mass spectrometry (IP-MS) analysis for Flag-ANKRD1 in Mahlavu	This paper	
LC-MS/MS analysis for siANKRD1 in Mahlavu	This paper	
Experimental models: cell lines		
Human: Mahlavu	Prof. Hui Kam Man (National Cancer Centre Singapore)	
Human: HLE	Prof. Hui Kam Man (National Cancer Centre Singapore)	
Human: HCCLM3	China Center for Culture Collection	
Human: SNU-886	Prof. Hui Kam Man (National Cancer Centre Singapore)	
Human: Huh1	Prof. Hui Kam Man (National Cancer Centre Singapore)	
Human: Huh7	China Center for Culture Collection	
Human: HepG2	China Center for Culture Collection	
Human: Hep3B	China Center for Culture Collection	
Human: HCC-ZJ	Our lab [9]	
Human: SNU-387	China Center for Culture Collection	
Human: SNU-449	Meisen Chinese Tissue Culture Collections	

Reagent and resource	Source	Cat#
Human: SNU-878	Meisen Chinese Tissue Culture Collections	
Mice: Hep-53.4	Meisen Chinese Tissue Culture Collections	
Human: LX-2	China Center for Culture Collection	
Oligonucleotides		
Human-*GAPDH*-RT-PCR-F: CGCTCACTGTTCTCTCCCTC<br>Human-*GAPDH*-RT-PCR-R: AAATGAGCCCCAGCCTTCTC	This paper	N/A
Human-*ANKRD1*-RT-PCR-F: AGTAGAGGAACTGGTCACTGG<br>Human-*ANKRD1*-RT-PCR-R: TGGGCTAGAAGTGTCTTCAGAT	This paper	N/A
Human-*TIMP1*-RT-PCR-F: CAATTCCGACCTCGTCATCA<br>Human-*TIMP1*-RT-PCR-R: ACGCTGGTATAAGGTGGTCT	This paper	N/A
Human-*TIMP2*-RT-PCR-F: TATCTCATTGCAGGAAAGGC<br>Human-*TIMP2*-RT-PCR-R: GAAGTCACAGAGGGTGATGT	This paper	N/A
Human-*ACTA2*-RT-PCR-F: CGTGGCTATTCCTTCGTTAC<br>Human-*ACTA2*-RT-PCR-R: TGCCAGCAGACTCCATCC	This paper	N/A

Reagent and resource	Source	Cat#
Human-*CCN2*-RT-PCR-F: AATGCTGCGAGGAGTGGGT Human-*CCN2*-RT-PCR-R: CGGCTCTAATCATAGTTGGGTCT	This paper	N/A
Human-*LOX*-RT-PCR-F: GTTCCAAGCTGGCTACTC Human-*LOX*-RT-PCR-R: GGGTTGTCGTCAGAGTAC	This paper	N/A
Human-*COL1A1*-RT-PCR-F: GGAATGAAGGGACACAGAGG Human-*COL1A1*-RT-PCR-R: TAGCACCATCATTTCCACGA	This paper	N/A
Human-*COL3A1*-RT-PCR-F: AGGACTGACCAAGATGGGAA Human-*COL3A1*-RT-PCR-R: AGGGGAGCTGGCTACTTCTC	This paper	N/A
Human-*COL6A1*-RT-PCR-F: CACTCAAAAGCAGCGTGGAC Human-*COL6A1*-RT-PCR-R: GTCGGTCACCACAATCAGGT	This paper	N/A
Human-*YAP1*-RT-PCR-F: ACCCACAGCTCAGCATCTTC Human-*YAP1*-RT-PCR-R: GACGTTCATCTGGGACAGCA	This paper	N/A

Reagent and resource	Source	Cat#
Human-*TAZ*-RT-PCR-F: CATGGCAGTATCCCAGCCAA Human-*TAZ*-RT-PCR-R: GGATTCTCTGAAGCCGCAGT	This paper	N/A
siANKRD1#1-sense: GGUCACUGGAAAGAAGAAU siANKRD1#1-antisense: AUUCUUCUUUCCAGUGACC	This paper	N/A
siANKRD1#2-sense: CGAUGGAUCUGGUGCUACA siANKRD1#2-antisense: UGUAGCACCAGAUCCAUCG	This paper	N/A
sgRNA-1: CATCTCTGAAATCCTCAGGAAGG	This paper	N/A
sgRNA-2: CTAGCCCACCCTGTGACCCTGGG	This paper	N/A
Human-*ANKRD1*-KOPCR-F: CTGCGGCATTGTCTGTGAAGAATG Human-*ANKRD1*-KOPCR-R: GAGACATCTTAATGATTGGGT	This paper	N/A
Mice-*Abcb4*-KOPCR-F1: AGCAGTCTCCCTATAGAACAACTCG Mice-*Abcb4*-KOPCR-R1: GCCTGGAACAGAATCAGAATATGTG	This paper	N/A

Reagent and resource	Source	Cat#
Mice-*Abcb4*-KOPCR-F2: TTTACAGCAACCAAGGCAGAG Mice-*Abcb4*-KOPCR-R2: TTAACCTCCCTGAGAGTAGTGAGC	This paper	N/A
Human-*ANKRD1*-ChIP-qPCR-F1: TCAGCAGGGTTAGCTTGTCC Human-*ANKRD1*-ChIP-qPCR-R1: TGTTTTTCTGTCGTGGAAGG	This paper	N/A
Human-*ANKRD1*-ChIP-qPCR-F2: TGCTAATTATGGCCAGTGACA Human-*ANKRD1*-ChIP-qPCR-R2: GTCCCAGGACATCCTATTCC	This paper	N/A
Human-*ANKRD1*-ChIP-qPCR-F3: TCAAGGGGGAAGAGGAGAAT Human-*ANKRD1*-ChIP-qPCR-R3: CGGAAGGGTTGAAAAAGGTC	This paper	N/A
Human-*ANKRD1*-ChIP-qPCR-F4: CTCTGTTTGCTTCAGCAGGA Human-*ANKRD1*-ChIP-qPCR-R4: AGGAAATCTCCTGGGCTTTT	This paper	N/A
siYAP1-sense: GCAUCUUCGACAGUCUUCU siYAP1-antisense: AGAAGACUGUCGAAGAUGC	This paper	N/A
siTAZ-sense: ACGUUGACUUAGGAACUUU	This paper	N/A

Reagent and resource	Source	Cat#
siTAZ-antisense: AAAGUUCCUAAGUCAACGU	This paper	N/A
Recombinant DNA		
pLVX-Puro-ANKRD1-3‘Flag	This paper	N/A
pLVX-Puro-ANKRD1-5‘Flag	This paper	N/A
pLVX-Puro-PRMT5-3‘HA	This paper	N/A
pLVX-Puro-ANKRD1-3‘Flag-ΔN	This paper	N/A
pLVX-Puro-ANKRD1-3‘Flag-ΔM	This paper	N/A
pLVX-Puro-ANKRD1-3‘Flag-ΔC	This paper	N/A
pGL3-Basic-promoter	This paper	N/A
pRL-SV40	This paper	N/A
Software and algorithms		
Modifit	Verity Software House	https://www.vsh.com/products/mflt/
Trimmomatic	THE USADEL LAB	http://www.usadellab.org/cms/?page=trimmomatic
Hisat2	DaehwanKim Lab	https://github.com/DaehwanKimLab/hisat2
StringTie	The Center for Computational Biology at Johns Hopkins University	https://github.com/gpertea/stringtie
htseq-count	htseq	https://github.com/htseq/htseq
DESeq2	R package	https://github.com/thelovelab/DESeq2
PASER	Bruker Corporation	
R 4.5.1		https://www.r-project.org/
GraphPad	GraphPad Software, Inc.	https://www.graphpad.com/

ANKRD1. Ankyrin repeat domain protein 1; GAPDH. Glyceraldehyde-3-phosphate dehydrogenase; PRMT5. Protein arginine methyltransferase 5; HA. Hemagglutinin;

HNRNPA0. Heterogeneous nuclear ribonucleoprotein A0; YAP. Yes1 associated transcriptional regulator; TAZ. Tafazzin; HRP. Horseradish peroxidase; HCC. Hepatocellular carcinoma; EdU. 5-ethynyl-2'-deoxyuridine; ChIP. Chromatin immunoprecipitation; PI. Propidium iodide; FITC. Fluorescein isothiocyanate; RNA-seq. RNA sequencing; IP. Immunoprecipitation; IF. Immunofluorescence; WB. Western blotting; IHC. Immunohistochemistry; MS. Mass spectrometry; LC. Liquid chromatography; COL6A1. Collagen type VI alpha 1 chain; TIMP2. TIMP metallopeptidase inhibitor 2; COL1A1. Collagen type I alpha 1 chain; COL3A1. Collagen type III alpha 1 chain; ACTA2. Actin alpha 2, smooth muscle; CCN2. Cellular communication network factor 2; LOX. Lysyl oxidase; *Abcb4*. ATP-binding cassette subfamily B member 4; ΔC. C-terminal truncation; ΔM. Middle region-truncation; ΔN. N-terminal truncation

References

1 Huang J, Xu L, Chen Y, Sun M, Hu Y, Pan C, *et al*. Redistribution of super-enhancers promotes malignancy in human hepatocellular carcinoma. J Adv Res. 2026;82:803-19. https://doi.org/10.1016/j.jare.2025.07.006.

2 Bolger AM, Lohse M, Usadel B. Trimmomatic: a flexible trimmer for Illumina sequence data. Bioinformatics. 2014;30(15):2114-20. https://doi.org/10.1093/bioinformatics/btu170.

3 Kim D, Langmead B, Salzberg SL. HISAT: a fast spliced aligner with low memory requirements. Nat Methods. 2015;12(4):357-60. https://doi.org/10.1038/nmeth.3317.

4 Pertea M, Pertea GM, Antonescu CM, Chang TC, Mendell JT, Salzberg SL. StringTie enables improved reconstruction of a transcriptome from RNA-seq reads. Nat Biotechnol. 2015;33(3):290-5. https://doi.org/10.1038/nbt.3122.

5 Anders S, Pyl PT, Huber W. HTSeq--a Python framework to work with high-throughput sequencing data. Bioinformatics. 2015;31(2):166-9. https://doi.org/10.1093/bioinformatics/btu638.

6 Love MI, Huber W, Anders S. Moderated estimation of fold change and dispersion for RNA-seq data with DESeq2. Genome Biol. 2014;15(12):550. https://doi.org/10.1186/s13059-014-0550-8.

7 Shen S, Park JW, Lu ZX, Lin L, Henry MD, Wu YN, *et al.* rMATS: robust and flexible detection of differential alternative splicing from replicate RNA-Seq data. Proc Natl Acad Sci U S A. 2014;111(51):E5593-601. https://doi.org/10.1073/pnas.1419161111.

8 Huang J, Xu L, Chen Y, Sun M, Hu Y, Pan C, *et al*. Redistribution of super-enhancers promotes malignancy in human hepatocellular carcinoma. J Adv Res. 2026;82:803-19. https://doi.org/10.1016/j.jare.2025.07.006.

9 Huang J, Sun M, Wang M, Yu A, Zheng H, Bu C, *et al*. Establishment and characterization of a highly metastatic hepatocellular carcinoma cell line. Bioengineered. 2024;15(1):2296775. https://doi.org/10.1080/21655979.2023.2296775.
